# Design and Evaluation of New 6-Trifluoromethoxy-Isatin Derivatives as Potential CDK2 Inhibitors

**DOI:** 10.3390/ijms27041802

**Published:** 2026-02-13

**Authors:** Przemysław Czeleń, Beata Szefler

**Affiliations:** Department of Physical Chemistry, Faculty of Pharmacy, Collegium Medicum, Nicolaus Copernicus University, Kurpinskiego 5, 85-096 Bydgoszcz, Poland; beatas@cm.umk.pl

**Keywords:** 6-Trifluoromethoxy-Isatin, CDK2, competitive inhibition, computational chemistry, molecular dynamics, ADMET

## Abstract

Cyclin-dependent kinase 2 (CDK2) plays a central role in cell cycle regulation and represents an important molecular target in anticancer drug development. In this study, a series of novel isatin derivatives substituted with a trifluoromethoxy group at the C6 position were designed and evaluated as potential CDK2 inhibitors using a comprehensive in silico approach. Density functional theory calculations were applied to analyze the electronic properties of the proposed compounds. Molecular docking and molecular dynamics simulations were used to investigate binding modes, conformational stability, and key interactions within the CDK2 active site. Binding free energies were estimated using the Molecular Mechanics Poisson–Boltzmann Surface Area (MMPBSA) method, while QSAR-based (Quantitative Structure–Activity Relationship) ADMET (Absorption, Distribution, Metabolism, Excretion, and Toxicity) analyses were performed to assess drug-likeness and pharmacokinetic profiles. The results indicate that the investigated derivatives form stable complexes with CDK2, supported by persistent hydrogen bonds in the hinge region and favorable hydrophobic interactions. The trifluoromethoxy substituent significantly affects ligand orientation and promotes deeper insertion into the hydrophobic pocket compared with previously studied isatin analogues. ADMET predictions suggest generally favorable absorption and toxicity profiles, with moderate solubility limitations. Overall, these findings support the potential of 6-trifluoromethoxy-isatin derivatives as promising CDK2 inhibitors and provide a basis for further experimental studies.

## 1. Introduction

Cancer continues to represent a substantial global health burden and remains one of the leading causes of death worldwide [1,2]. Despite significant advances in diagnostic techniques and the development of new therapeutic strategies, effective control and prevention of this group of diseases remain limited. A defining feature of cancer is uncontrolled cellular proliferation, which arises from disruptions in the tightly regulated mechanisms governing the cell cycle. Central to the regulation of these processes are enzymes belonging to the cyclin-dependent kinase family, which are involved in the control of cell cycle progression, transcriptional regulation, and DNA synthesis [3,4,5]. Among these enzymes, cyclin-dependent kinase 2 (CDK2) plays a pivotal role in regulating the transition from the G1 to the S phase, as well as progression through the S phase. Numerous studies have demonstrated that dysregulation, hyperactivation, or overexpression of CDK2 is frequently observed across a wide range of cancer types and is strongly linked to excessive proliferation, impaired apoptotic signaling, and genomic instability [6,7,8,9,10]. These features make CDK2 a compelling molecular target in anticancer drug development.

Although several CDK inhibitors have successfully entered clinical use, their application is often constrained by insufficient selectivity, off-target effects, and toxicity-related dose limitations [7,11,12,13]. The high degree of structural conservation within the ATP-binding pockets of CDK family members further complicates the development of selective inhibitors. As a result, there is a continuing need to identify new chemical frameworks capable of achieving stable, selective, and effective interactions with the CDK2 active site.

In this context, isatin (1H-indole-2,3-dione) has emerged as a valuable and pharmacologically relevant scaffold in medicinal chemistry. The oxindole core of isatin is particularly well suited for kinase inhibition due to its ability to establish key interactions within the ATP-binding region, especially in the hinge area of CDK2 defined by residues such as GLU81 and LEU83 [14]. Previous investigations have shown that strategic structural modifications of the isatin framework—most notably substitutions on the aromatic ring and functionalization at the C-3 position—can substantially improve binding affinity, inhibitory potency, and overall stability of ligand–kinase complexes [15,16,17,18].

Recent studies further underscore the significance of isatin-derived compounds in the development of cyclin-dependent kinase inhibitors, with particular emphasis on CDK2. Comprehensive structure–activity relationship analyses have demonstrated that isatin-based scaffolds, especially those functionalized at the C-3 position, can achieve high inhibitory potency and selectivity toward CDK2 through effective hinge-region interactions and stabilization within the ATP-binding pocket [19]. Moreover, isatin derivatives bearing hydrazide or hydrazone side chains—representing close structural analogues of the compounds discussed herein—have been reported to exhibit pronounced antiproliferative activity and kinase inhibition profiles [20,21,22,23,24]. The presence of hydrazide-linked moieties was shown to enhance hydrogen-bonding networks and conformational flexibility, facilitating improved accommodation within the CDK2 catalytic cleft. Collectively, these findings highlight isatin-based frameworks as a versatile and pharmacologically relevant platform for the rational design of CDK2 inhibitors and provide strong justification for further exploration of structurally related derivatives.

Our research group has previously focused on the rational design of isatin-based CDK2 inhibitors through a combination of computational and experimental methodologies. Approaches including quantum-mechanical calculations, molecular docking, molecular dynamics simulations, and ADMET prediction have been applied to optimize molecular properties relevant to both activity and pharmacokinetics. These studies highlighted the critical influence of substituent type and position on ligand orientation, hydrogen-bond formation, and penetration into the hydrophobic pocket of CDK2. In particular, compounds bearing electron-withdrawing groups or moieties capable of forming additional stabilizing interactions exhibited superior binding characteristics compared with known reference inhibitors [15,17,25].

Extending these findings, further diversification of the isatin scaffold appears to be a promising strategy toward improved CDK2 inhibition. Previous studies on isatin derivatives bearing nitro or methyl substituents at the 5-position [15,17] demonstrated that such modifications play a key role in modulating interactions of the isatin core by enforcing an alternative orientation relative to the active site and promoting additional intermolecular contacts. In this context, particular attention has been directed toward fluorinated substituents, as fluorine-containing groups are well recognized for their ability to modulate electronic distribution, lipophilicity, metabolic stability, and intermolecular interactions. Accordingly, incorporation of a trifluoromethoxy group at the 6-position of the isatin core may exert an even more pronounced effect, contributing more substantially to ligand orientation within the CDK2 binding pocket and to the formation of stabilizing interactions, thereby potentially enhancing inhibitory activity.

Based on this rationale, the present study focuses on the design and in silico evaluation of a new series of 6-trifluoromethoxy isatin derivatives as potential CDK2 inhibitors. An integrated computational strategy encompassing molecular docking, molecular dynamics simulations, MMPBSA binding free energy calculations, QSAR analysis, and ADMET profiling was employed to investigate binding affinity, structural stability, and drug-likeness of the proposed compounds. The obtained results provide deeper insight into the structure–activity relationships of isatin-based CDK2 inhibitors and further support the suitability of this scaffold for the development of novel anticancer therapeutics.

## 2. Results and Discussion

**Figure 1:**
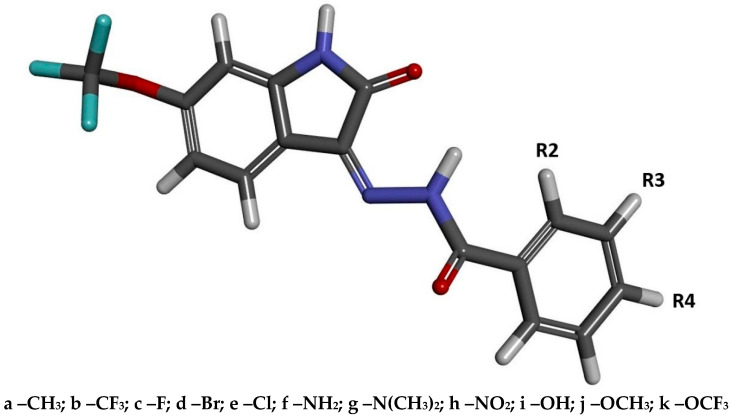
The graphical representation of 6-Trifluoromethoxyisatin-based benzoylhydrazines. The markings R2, R3, and R4 represent the places of substitution of chemical groups. Symbolic designation of the synthesized 6-trifluoromethoxyisatin-based benzoylhydrazine derivatives, assigned according to the type and positional location of the substituent on the benzoylhydrazine ring. For example, compound 2a contains a methyl group at the R2 position.

The group of compounds investigated in the present study belongs to the class of isatin derivatives, for which significant inhibitory activity against cyclin-dependent kinases (CDKs) has been repeatedly reported. The isatin molecular scaffold offers broad opportunities for the development of new classes of compounds through modifications introduced directly into the core structure, particularly at the fifth and sixth positions, as well as through reactions enabling the attachment of new moieties within the pyrrolidinone ring, for example via reactions with benzoylhydrazide derivatives. This approach, previously described in our earlier work [17], allows for the design of compounds with an improved fit within the active-site cavity and enhanced binding affinity, ultimately resulting in increased inhibitory potential. The model structure illustrating the geometry of the new group of inhibitors is shown in Figure 1, with labels R2, R3, and R4 indicating the potential positions of substituents on the benzoylhydrazide aromatic system. Based on commercially available substituted benzoylhydrazide derivatives, 34 new compounds were designed, and their inhibitory and pharmacological potential was evaluated using a wide range of computational chemistry methods.

### 2.1. Frontier Orbitals Analysis

The electronic properties of the designed group of compounds were investigated using DFT methods. The frontier orbital energies and derived parameters characterizing the potential reactivity of the studied inhibitors are presented in Table 1 and Table S2. The HOMO energies range from −6.5 eV (compounds 1, 2a, 2c, 2i, and the series of derivatives with substituents at position 3) to −5.9 eV (2f), while derivatives with substituents at position 4 exhibit intermediate values ranging from −6.0 eV to −6.2 eV. The higher HOMO energies observed for compounds 2f and 4f indicate an increased ability to donate electron density, which favors donor interactions with electrophilic active groups located in the CDK2 active site, such as LYS33 and LEU83. The LUMO energies fall within a narrow range of −2.4 to −2.6 eV, with the lowest values observed for structures 3h, 3k, 4b, and 4d, suggesting their potentially greater electron-accepting ability and slightly enhanced capacity to stabilize charge-transfer interactions with nucleophilic groups present in amino acid residues such as ASP145, GLU51, and GLU81.

The localization of the HOMO and LUMO orbitals of the studied compounds is presented in Figure S1 (Supplementary Materials). For most of the compounds, the localization of both types of orbitals exhibits a normalized and consistent distribution. Exceptions are derivatives containing strongly electron-donating substituents, such as –NH_2_ (2f, 4f) or mie–OH (2i, 4i), which significantly contribute to enhanced reactivity of the benzoylhydrazide moiety.

The energy gap (ΔEGAP) is a key descriptor reflecting the electronic polarizability and intramolecular charge-transfer capability of the studied compounds. Within the investigated ligand set, ΔE_GAP_ values range from 3.53 to 4.04 eV. Higher values, observed for structures 1, 2a, 2c, 2i, and 3a–3e, indicate slightly lower polarizability and greater kinetic stability, features that may favor more selective and structurally well-defined inhibitory binding. Conversely, derivatives 2f, 3k, and compounds from series 4 exhibit lower ΔE_GAP_ values, suggesting enhanced electronic flexibility and a greater propensity for polarization-driven interactions within the enzyme active site, including π–π contacts with aromatic residues such as PHE80 and PHE82.

The reported values of chemical hardness (η) fall within a relatively narrow range of 1.77–2.02 eV. Lower hardness values, accompanied by higher softness values (σ = 0.55, S ≈ 0.27), indicate that structures such as 2f, 3k, and compounds from series 4 may exhibit a slightly greater ability to adapt to the properties of the investigated active site.

The absolute electronegativity (χ) determined for the studied compounds ranges from 4.18 to 4.57 eV. Compounds with higher absolute electronegativity values exhibit a stronger electron-accepting character, which may represent a favorable factor contributing to stabilization of the inhibitor–active-site complex through stronger electrostatic interactions and charge transfer. The global electrophilicity index (ω) reaches the highest values for ligands 3h (5.30 eV), 3b–3e (5.11 eV), and 2c (5.01 eV). These values predispose these ligands to greater efficiency in interactions with nucleophilic amino acid residues within the CDK2 active site, potentially enhancing the stability of the formed complexes. Lower values of this parameter observed for ligands 2i (4.89 eV) and 4f (4.84 eV) indicate a more balanced distribution of donor–acceptor properties and potentially more diverse modes of interaction with the active site.

### 2.2. Molecular Docking

**Figure 2:**
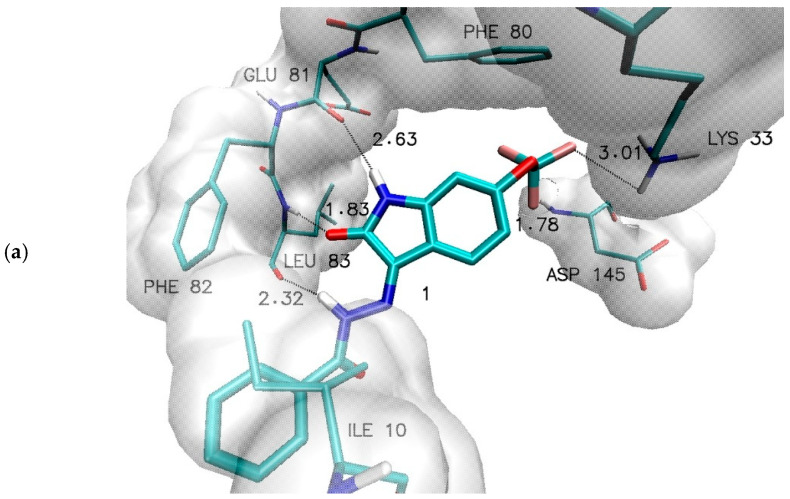

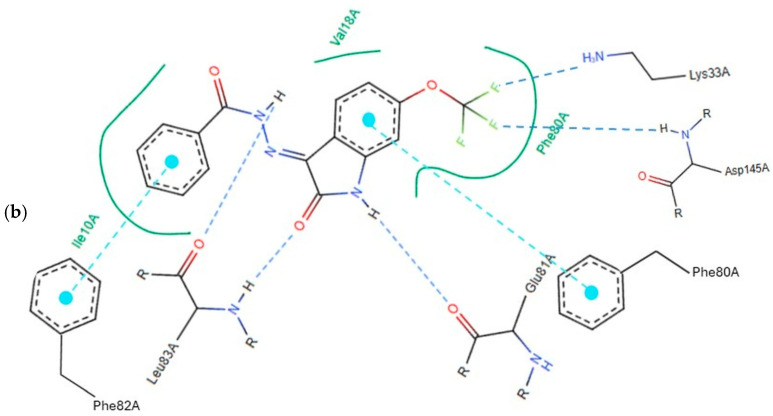
The graphic representation of N′-[6-Trifluoromethoxy-2-oxo-1,2-dihydro-3H-indol-3-ylidene]benzohydrazide (1) complex with active site of CDK2 enzyme 3D (**a**) and 2D (**b**).

For the purposes of this study, 34 novel 6-trifluoromethoxyisatin derivatives were designed, including the native form and 33 derivatives bearing substituents within the benzoylhydrazide ring. Based on these compounds, a molecular docking procedure was performed to obtain the most representative conformations of complexes formed between the selected inhibitors and the active site of cyclin-dependent kinase 2 (CDK2). A representative complex formed by the native derivative is shown in Figure 2. The ligand core is tightly embedded within the hinge region of the protein, in close proximity to GLU81 and LEU83. Notably, the trifluoromethoxy group also participates in binding interactions, with fluorine atoms forming hydrogen bonds with ASP145 and LYS33. An important stabilizing factor of the resulting complex is provided by hydrophobic interactions, in which aromatic systems of amino acid residues located in this region (PHE80, PHE82), as well as extended aliphatic groups such as that of ILE10, play a significant role.

The chemical affinity and inhibition constants (IC) describing the binding capabilities of each considered ligand toward the CDK2 active site are presented in Table 2. The IC values were obtained based on the Van’t Hoff Equation (1).

(1) $$IC={exp}^{\left(\frac{{\Delta G}_{b}}{RT}\right)}$$

The presented dataset allows for the characterization of changes in the binding capabilities of the studied ligands in relation to the presence and nature of substituents located at the R2, R3, and R4 positions. The binding affinities of the modified derivatives were evaluated relative to the reference value of −9.80 kcal/mol obtained for the native molecule lacking any substituents on the aromatic system.

For modifications located at the R2 position, the greatest diversity of observed effects is evident. The introduction of sterically bulky substituents, which may constitute significant steric hindrance—as observed for derivatives 2g, 2h, 2i, 2j, and 2d—results in a decrease in binding affinity, with the most pronounced reduction exceeding 1.5 kcal/mol. In contrast, derivatives 2a, 2c, 2f, and 2i exhibit improved binding capabilities; however, the observed increases remain within the range of −0.1 to −0.2 kcal/mol.

Within the group of derivatives bearing substituents at the R3 position, no structure was identified in which the presence of a modification negatively affected binding affinity relative to the native compound. An increase in affinity was observed for derivatives 3a, 3b, 3c, 3d, 3e, 3h, and 3k, with gains ranging from −0.1 to −0.2 kcal/mol.

The final group comprises derivatives modified at the R4 position. Within this population, three compounds exhibit reduced binding affinity compared to the native molecule, while seven derivatives show enhanced binding activity. Consistent with the trends observed for the other substitution patterns, the enhancements in binding affinity within this group remain modest and do not exceed −0.2 kcal/mol.

Analysis of the effects of individual substituents indicates that the introduction of groups such as –CH_3_, –CF_3_, and –F significantly enhances the binding capabilities of the investigated derivatives, regardless of their position within the benzoylhydrazide ring.

Within the investigated group, 18 derivatives can be identified for which the introduction of structural modifications resulted in enhanced binding affinity toward the active site of cyclin-dependent kinase 2 (CDK2). The properties of these complexes were analyzed in greater detail relative to those of the system formed by the native molecule. Table 3 presents parameters describing hydrogen-bond lengths as well as distances between aromatic systems involved in hydrophobic interactions.

The most critical interactions in the context of previous studies on isatin derivatives binding within the hinge region of CDK2 are hydrogen bonds formed with GLU81 and LEU83. For the investigated derivatives, the hydrogen bond involving the hydrogen atom of LEU83 falls within a narrow range of 1.73–1.90 Å, classifying these interactions as strong hydrogen bonds. Another key interaction involves the oxygen atom of GLU81; for most of the studied derivatives, the corresponding bond length is approximately 2.63 Å. An exception is derivative 2a, for which a slightly shorter distance of 2.36 Å was observed. These values indicate the presence of weak hydrogen bonds.

An additional interaction, not involving the isatin core, engages a hydrogen atom from the hydrazine moiety of the side chain and the oxygen atom of LEU83, with observed distances ranging from 2.25 to 2.59 Å. Another group of hydrogen bonds was identified for fluorine atoms of the trifluoromethoxy group located at the 6-position of the isatin core. This group participates in the formation of two hydrogen bonds: the first with ASP145, which for most of the analyzed systems exhibits distances between 1.76 and 1.78 Å, with exceptions observed for derivatives 2a (1.69 Å) and 3b (2.23 Å). The second interaction involves hydrogen atoms of the amino group of LYS33, with bond lengths predominantly ranging from 2.38 to 2.52 Å; an exception is structure 1, for which a distance of 3.01 Å was measured, representing a borderline value for hydrogen bonding.

Another class of interactions that is particularly important for stabilizing ligand–active-site complexes comprises hydrophobic interactions. These were quantified by measuring distances between aromatic systems capable of forming so-called stacking interactions, characteristic of aromatic moieties. In most cases, the interaction between the isatin core and the aromatic system of the PHE80 residue is described by distances slightly exceeding 4 Å. A notably shorter distance was observed only for derivative 2a (3.74 Å), for which the hydrogen-bond lengths involving LEU83 and GLU81 also indicate deeper embedding of this part of the molecule within the hydrophobic pocket compared to the remaining structures.

The final set of parameters related to hydrophobic interactions includes distances defined between the aromatic system of PHE82 and the aromatic moiety of the ligand side chain. The observed values range from 3.69 to 4.23 Å and indicate greater conformational freedom of this ligand fragment, resulting from the architecture of the active site and the nature of substituents present on the aromatic system of the analyzed derivatives. Overall, all reported distances fall within ranges compatible with the occurrence of stacking interactions between the ligand and active-site amino acid residues, while the dispersion of values reflects differences in the strength of these interactions.

**Figure 3:**
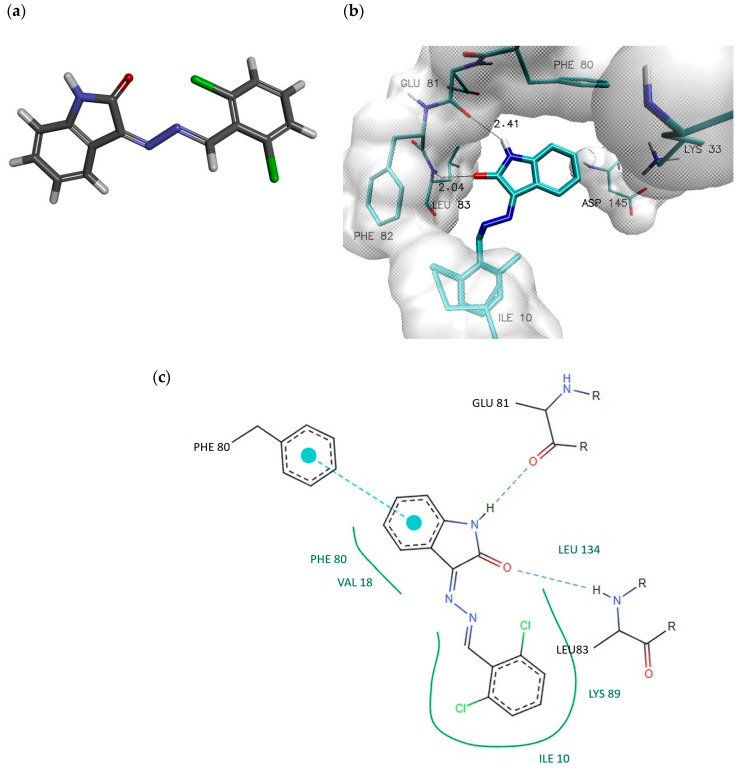
The graphical representation of reference molecule (RM) 3-((2,6-Dichlorobenzylidene)hydrazono)indolin-2-one (**a**). The graphical representation of RM complex with active site of CDK2 enzyme 3D (**b**) and 2D (**c**) obtained during docking stage.

A proper evaluation of the inhibitory potential of the proposed compounds requires comparison of the obtained results with data for systems exhibiting confirmed and experimentally validated inhibitory activity. Therefore, 3-((2,6-dichlorobenzylidene)hydrazono)indolin-2-one was selected as the reference molecule (RM). In vitro studies have clearly demonstrated that this compound exhibits inhibitory activity against CDK2. The activity of the reference molecule toward the CDK2 active site was described in a previous study [15], and the structure of the reference compound as well as its complex with the CDK2 active site are presented in Figure 3.

The chemical structure of the reference molecule exhibits significant structural similarity to the studied group of inhibitors. In both cases, isatin served as the starting scaffold for compound design; however, the distinguishing features include a trifluoromethoxy group substituted at position 6 and differences in the nature of the substituent attached to the side chain. Docking studies performed for the reference molecule indicated a markedly lower affinity toward the investigated active site. The obtained binding energy value of −8.62 kcal/mol [15] suggested weaker binding not only in comparison with the most active derivatives (−10.1 kcal/mol) but also relative to the native model structure (−9.8 kcal/mol).

The localization of the isatin core within the active-site space was generally consistent with the conformations observed for the studied group of compounds; however, noticeable differences in its orientation were evident. These differences were reflected in variations in the interaction distances with GLU81 and LYS83, as well as in the distance between PHE80 and the aromatic system of the core. In contrast, the orientation of the aromatic moiety in the side chain differed substantially, with distances measured between this moiety and PHE82 being at least 1 Å longer. Based on the observed differences in binding affinity and the identified interactions, it could be concluded that the proposed compounds may exhibit significantly greater potential for stabilizing interactions within the active site of the biological target.

### 2.3. Molecular Dynamics

All ligand structures for which an increase in binding affinity toward the active site was observed compared to the native structure were selected for the molecular dynamics simulation stage. One of the most fundamental yet critically important evaluations of systems obtained from molecular dynamics simulations is the assessment of their conformational stability and its variation over the temporal evolution of the studied systems. The analysis of ligand mobility and conformational variability provides a general indication of the correctness of inhibitor fitting within the active site at the docking stage and allows for evaluation of the stability of the formed complexes. Table 4 presents a set of data illustrating the averaged RMSD values and their standard deviations for the analyzed populations, considering both the entire simulation time and the final 60 ns of the trajectories. This distinction was introduced because the analysis of the time-dependent distributions of the studied systems (Figure S2) indicated that the initial 20 ns of the simulations were, in most cases, associated with significant conformational changes manifested by pronounced RMSD fluctuations. A superficial comparison of mean values and standard deviations might suggest that this issue mainly concerns the conformational properties of the active site; however, statistical testing based on one-way analysis of variance (ANOVA) for paired datasets revealed statistically significant differences between the compared populations for eleven ligands. In contrast, for parameters describing the active site, statistical consistency between conformer populations collected over 80 ns and 60 ns was observed in only one case (compound 3a). Therefore, based on these observations, subsequent analyses focusing on interaction characterization and binding affinity were restricted to the final 60 ns of the molecular dynamics simulations.

The analysis of the time evolution of ligand RMSD values, presented in Figure S2, clearly indicates that several groups of ligands exhibiting distinct conformational stability characteristics can be distinguished within the studied population. Some structures display significant conformational changes throughout the entire molecular dynamics production phase, cyclically affecting their structural properties. This behavior is observed for compounds 2c and 3c bearing fluorine substituents, which exhibit the most pronounced and relatively chaotic conformational fluctuations.

More ordered conformational transitions were observed for derivative 3b containing a trifluoromethyl substituent; in this case, the recorded increase in RMSD was associated with a 180° rotation of the aromatic moiety in the side chain. In contrast to derivatives 2c and 3c, this conformational change was a single event, and the adopted conformation remained stable thereafter.

Another group consists of systems that undergo relatively dramatic conformational changes during the initial 20 ns of the simulation but subsequently achieve a stable conformational arrangement within the active-site space, as observed for derivatives 3a, 3h, and 4a.

The final and most numerous group comprises molecules that underwent conformational rearrangements during the initial optimization stage relative to the starting geometry and then exhibited only minor oscillations throughout the molecular dynamics simulations. Representative examples of this behavior include derivatives 1, 2a, 2i, 3e, 4b, 4g, and 4i. Overall, the RMSD values clearly indicate that, over the course of the molecular dynamics simulations, each ligand underwent subtle conformational adjustments aimed at optimizing its fit within the active site.

The stability of complexes formed by ligands with the active sites of the investigated biological targets is governed by the presence of a broad spectrum of interactions, including electrostatic and van der Waals interactions, hydrogen bonds, halogen bonds, as well as a wide range of hydrophobic interactions, including, among others, π–π stacking interactions. In order to unambiguously verify the stability of the studied complexes, a detailed analysis of hydrogen bonds identified at the docking stage as well as those formed during molecular dynamics simulations was performed. A summary of the cumulative values for individual hydrogen bonds is presented in Table 5 and Table S3.

For all analyzed compounds, several general trends can be observed with respect to both the number and quality of the formed hydrogen bonds. Among all interactions identified during the docking phase, only two hydrogen bonds retained full stability and persistence in the conformers sampled during molecular dynamics simulations: Ligand (H1)···(O) GLU81 and Ligand (O1)···(HN) LEU83. These interactions were present in 100% of the conformers obtained during the molecular dynamics simulations; however, significant changes in their bond lengths were observed compared to those identified at the docking stage, indicating a reorientation of the isatin core relative to the hinge region of the protein comprising LEU83 and GLU81.

The observed discrepancies are manifested by a pronounced shortening of the hydrogen bond formed with GLU81, which initially oscillated around 2.6 Å in the docked complexes, whereas in the vast majority of conformers (≥80%) its length ranged from 1.6 to 2.0 Å. The second key hydrogen bond involving LEU83 also exhibited slight changes in its properties. The originally identified distance of approximately 1.8 Å was observed, depending on the ligand, in only 26–40% of the analyzed conformers, while a considerably larger population of structures (~60%) exhibited distances in the range of 2.0–2.2 Å.

Much more pronounced changes were observed for the Ligand (H5)···(O) LEU83 hydrogen bond, whose significance nearly vanished in complexes formed by ligands 2a and 2c. For many derivatives, the presence of this interaction was detected in fewer than 50% of the conformers, and the recorded bond lengths indicated a dominant contribution of weak interactions or interactions merely fulfilling the boundary criteria for hydrogen bonding (compounds 1, 2f, 2i, 3c, 4c, 4d, 4e, and 4f). Conversely, complexes were also identified in which this hydrogen bond occurred with higher frequency (70–80%), with a substantial fraction of the population corresponding to hydrogen bonds of intermediate strength (compounds 3a, 3b, 3d, 3e, 3h, 4a, and 4b).

The aforementioned reorientation of the isatin core simultaneously influenced the ability of fluorine atoms from the trifluoromethoxy group located at position 6 of the central ring to participate in hydrogen bond formation. The strong hydrogen bond involving the hydrogen atom of ASP145 exhibited more diverse characteristics in the systems sampled during molecular dynamics simulations. No complex was identified in which this interaction was present in 100% of the conformers; nevertheless, several complexes demonstrated its significant contribution to complex stabilization. Notably, derivatives 3h and 4e exhibited this interaction in at least 95% of the analyzed conformers. Although the bond lengths increased noticeably compared to the initial docked structures, the dominant fraction of the population could still be classified as hydrogen bonds of moderate strength. A significant stabilizing role of the hydrogen bond formed with ASP145 was also observed in complexes formed by derivatives 1, 2i, 3b, 3c, 3d, 3k, 4a, 4d, and 4f, where the fraction of conformers confirming this interaction ranged from 70 to 85%.

The most labile hydrogen bond identified during the docking stage is the interaction formed with LYS33. Analysis of the collected conformational populations indicates that its occurrence ranges from less than 20% to approximately 46%, with the dominant contribution arising from bond lengths characteristic of weak hydrogen bonds. For the majority of the analyzed populations, the fraction of conformers confirming the presence of this interaction oscillates around 35%. Although this hydrogen bond may contribute to the overall spectrum of interactions stabilizing the complexes, it cannot be regarded as a dominant factor governing their formation.

In the case of the reference molecule, it can be observed that the hydrogen bond formed between GLU81 and the hydrogen atom of the isatin core exhibits characteristics similar to those identified for the newly proposed compounds. In contrast, significant differences are evident in the properties of the hydrogen bonds formed with the hydrogen atom of LEU83. For this interaction, only a negligible contribution of strong hydrogen bonds is observed (bond length range 1.6–1.8 Å, approximately 6%), whereas hydrogen bonds of intermediate strength constitute the dominant group, accounting for approximately 71% of the conformers.

**Figure 4:**
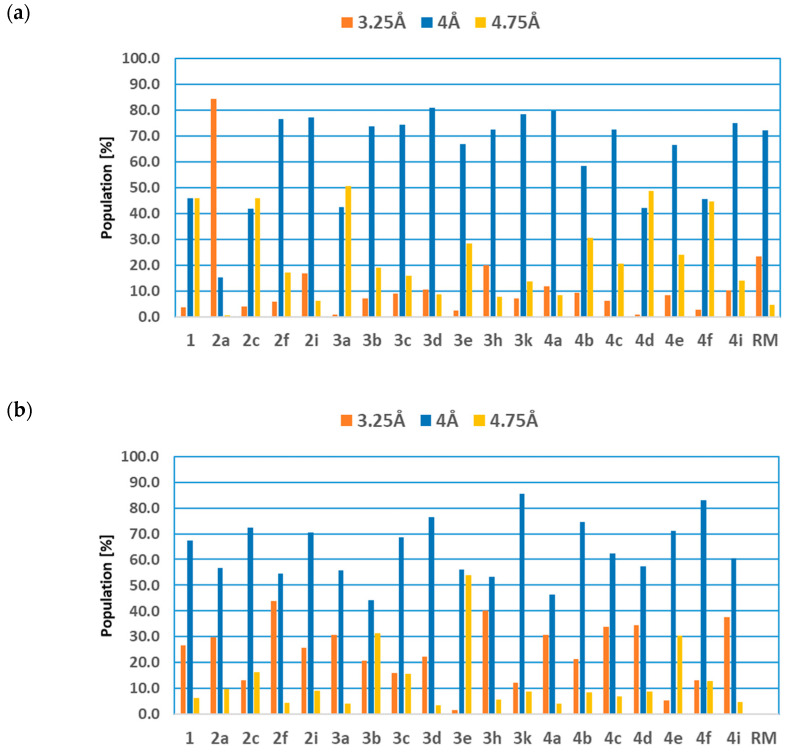
The cumulative analysis of the distances between aromatics systems of 6-Trifluoromethoxyisatin-based benzoylhydrazides and phenyloalanines PHE80 (**a**) and PHE 82 (**b**). The distances presented in chart labels represent middle values of intervals with a width of 0.75 Å.

Another important factor determining the stability of the studied complexes is the broad range of hydrophobic interactions. A key aspect of this type of interaction is π–π stacking between aromatic systems, the strength of which is primarily determined by the distance between the interacting aromatic rings. Figure 4 presents a cumulative analysis illustrating the distribution of distances between the aromatic system of PHE80 and the isatin core (Figure 4a), as well as between PHE82 and the aromatic ring located in the side chain of the studied ligands (Figure 4b).

In the case of the first interaction, it can be observed that for the majority of the analyzed systems, the dominant fraction (70–80% of conformers) corresponds to distances oscillating around 4.0 Å. This value correlates well with the distances identified at the docking stage, and a similar interaction profile is also observed for the reference molecule. A subset of derivatives exhibiting a significantly lower tendency for deep insertion of the molecular core into the hydrophobic pocket can also be identified, namely derivatives 1, 2c, 3a, 4d, and 4f.

An exceptional case within the analyzed group is derivative 2a, for which nearly 85% of the conformers exhibit distances between the aromatic systems oscillating around 3.25 Å. Such pronounced penetration of the molecular core into the hydrophobic pocket is further supported by the high fraction of very strong hydrogen bonds formed with GLU81 (approximately 63%).

For the second set of analyzed aromatic interactions, a relatively uniform profile is observed for most of the studied systems, characterized by a dominant population of conformers in which the distance between the aromatic systems fluctuates around 4.0 Å. A slightly different behavior is observed for derivatives 2f and 3h, where approximately 40% of the conformers display significantly shorter distances, close to 3.25 Å. The only derivative for which a pronounced increase in the distance between the aromatic systems relative to the initial docked structure is observed is compound 3e, for which more than half of the analyzed population exhibits distances oscillating around 4.75 Å.

A noteworthy and expected exception is the reference structure (RM), which, due to the presence of symmetrically distributed chloro substituents, exhibits limited ability to interact within the active-site region containing the aromatic system of PHE82.

**Figure 5:**
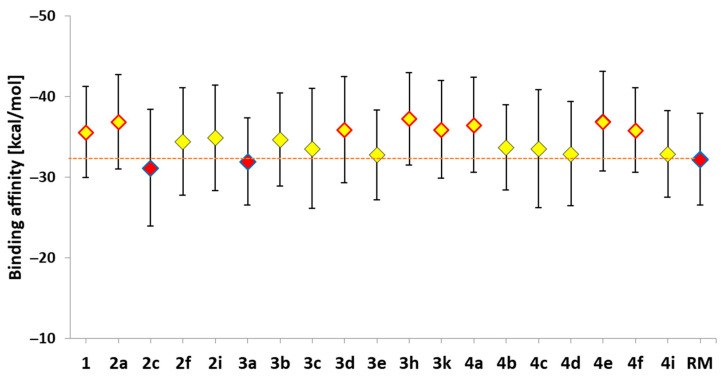
The values of enthalpic contribution to the binding affinity [kcal/mol] estimated for all complexes considered during molecular dynamics stage. For values marked with red indicators, statistical analysis based on one-way analysis of variance (ANOVA) with the pairwise multiple comparison Holm–Sidak method showed a statistically significant difference compared to values obtained for the native system (1). For markers within the red frame, a significant difference was observed relative to the reference molecule (RM).

The final stage of the molecular dynamics–based analyses, aimed at extending the characterization of the studied systems beyond structural stability and conformational variability of the active sites, was based on the Molecular Mechanics Poisson–Boltzmann Surface Area (MMPBSA) method. The calculations were performed using systems extracted from the final and most stable phase of the molecular dynamics simulations, covering the last 60 ns of the trajectories. The collected data, presenting averaged enthalpic contributions to binding affinity along with standard deviations, are shown in Figure 5. It can be observed that although a considerable population of conformers exhibited higher binding affinity values than the native ligand (i.e., 2a, 3d, 3h, 3k, 4a, 4e, and 4f), statistical analysis using one-way analysis of variance (ANOVA) with the pairwise multiple comparison Holm–Šidák method did not indicate the presence of statistically significant differences between the compared datasets.

Within the studied group, derivatives that showed statistically significantly lower binding affinities relative to the native system included compounds 2c, 3a, and the reference molecule (RM). In contrast, a substantially broader group of derivatives demonstrated statistically significant improvements in binding affinity relative to the reference molecule, including compounds 1, 2a, 3d, 3h, 3k, 4a, 4e, and 4f.

**Figure 6:**
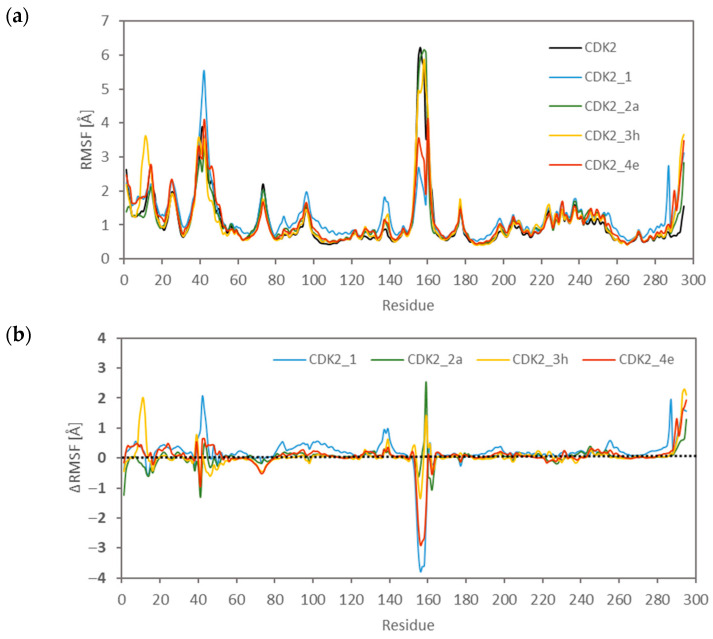
Graphical representation of the root mean square fluctuation (RMSF) values of CDK2 residues for the free and complexed protein (**a**). The second panel shows the differences between RMSF values of residues in the complexed and free protein (**b**).

Ligand–binding site interactions are inherently bidirectional; not only does the ligand undergo conformational adaptation to the active site, but the active site itself may also experience ligand-induced conformational changes. To assess this phenomenon, extended molecular dynamics simulations of 160 ns were performed for representative systems, including the apo protein as well as complexes with the native ligand and selected derivatives bearing substituents at the R2, R3, and R4 positions (2a, 3h, and 4e), which also exhibited the highest affinity toward the active site. Based on the collected trajectories, root mean square fluctuation (RMSF) values were calculated for individual amino acid residues comprising CDK2. A comparative summary of the RMSF profiles is presented in Figure 6a,b. The largest structural fluctuations of the analysed protein are observed in two residue ranges, namely 35–55 and 145–165. The former corresponds to a loop region and a fragment of an α-helix located in close proximity to the active site, whereas the latter represents the most flexible segment of the protein, namely the T-loop. More specific and less pronounced changes are observed for other residue ranges forming β-sheet elements that delimit the active-site cavity in the upper (residues 6–15, 20–30, and 70–85) and lower planes (residues 130–140).

The observed fluctuations clearly indicate that the presence of a bound ligand significantly influences the conformational properties of the active site and the structural dynamics of the amino acid residues forming this region.

### 2.4. ADMET Analysis

Assessment of the interaction potential of a candidate drug with its biological target constitutes a fundamental aspect of drug design; however, it is accompanied by other essential activities aimed at evaluating the overall molecular properties and in vivo behavior of the investigated substances. In this context, methods based on quantitative structure–activity relationship (QSAR) analysis are particularly useful. Such tools enable the evaluation of a broad spectrum of compound properties, including absorption, distribution, metabolism, excretion, and toxicity (ADMET) [26,27,28,29]. The selected set of descriptors characterizing the properties of the investigated group of compounds is presented in Table 6.

To facilitate a better interpretation of the ADMET parameters describing the studied group of compounds, a broader set of reference compounds was included in the analysis. This reference group comprises substances not only investigated in clinical studies but also widely used in the development of new and effective anticancer therapies, namely roscovitine (RM2) [30,31] and dinaciclib (RM3) [32], which are anticancer drugs acting as cyclin-dependent kinases (CDK’s) inhibitors.

One of the most basic parameters considered in this dataset is molecular weight (MW), which for the analysed population of compounds ranges from 363.08 to 433.05 u. All identified values fall below the commonly accepted threshold (MW < 500 u) [33]. The subsequent parameters describe the numbers of hydrogen bond donors (nHD) and hydrogen bond acceptors (nHA). In both cases, the values identified for the investigated compounds comply with the criteria defined for molecules with pharmacological potential [33].

Comparison of the aforementioned parameters for the reference molecule 1 (RM) and the investigated group of compounds reveals that the reference compound possesses a lower molecular weight and a lower degree of saturation with functional groups acting as hydrogen bond donors and acceptors. This trend is also reflected in another analyzed parameter, namely the topological polar surface area (TPSA) [34], for which the values recorded for the investigated compounds are significantly higher and, in several cases, nearly twice as large. The observed disparities in TPSA values may indicate differences in the ability of the studied compounds to permeate environments where lipophilicity is a critical factor. Nevertheless, this observation does not imply an inability of the investigated compounds in this regard, but rather suggests a relative advantage of the reference compound 1. When the parameter values obtained for the studied group of compounds are compared with those calculated for roscovitine (RM2) and dinaciclib (RM3), it can be observed that they are characterized by very similar numbers of hydrogen bond donors and acceptors, as well as comparable TPSA values, indicating a high degree of similarity in the overall physicochemical profiles of the compounds under comparison.

The subsequent parameters presented relate to the solubility of the investigated compounds, namely the octanol/water partition coefficient (logP) and water solubility (logD). The reported data indicate that, in most cases, the studied substances exhibit values that slightly exceed the optimal range recommended for pharmacologically active compounds; however, they remain well below the established threshold limits [33]. In the analysis of the solubility-related parameters of the studied compounds, it can be observed that although the investigated substances do not exhibit optimal solubility properties, in most cases they demonstrate more favorable characteristics than roscovitine (RM2). This suggests that the potentially lower solubility of the studied compounds should not significantly limit their applicability or therapeutic potential in anticancer treatments.

Issues associated with limited solubility in common solvents can be substantially mitigated through the use of binary solvent systems based on trace amounts of organic solvents, which are capable of significantly improving the solubility of the investigated substances, as demonstrated in studies conducted on analogous classes of isatin derivatives [25].

Based on the descriptors presented thus far, the properties of the investigated compounds can be evaluated using the most widely recognized drug-likeness criterion, namely Lipinski’s Rule of Five. This rule directly links the pharmacological potential of candidate compounds to compliance with the following guidelines: molecular weight (MW ≤ 500), logP (≤5), number of hydrogen bond acceptors (nHA ≤ 10), and number of hydrogen bond donors (nHD ≤ 5) [33]. The values presented unequivocally indicate that the proposed group of compounds satisfies all the requirements of this rule.

Another important set of parameters describes blood–brain barrier (BBB) permeability and clearly indicates that none of the proposed compounds is predicted to exhibit difficulties in crossing the barrier between the bloodstream and the central nervous system. An additional crucial aspect of drug absorption is its ability to be absorbed and permeate following oral administration. The reported values of the Caco-2 [35] and human intestinal absorption (HIA) parameters indicate that all proposed compounds exhibit favorable absorption properties upon oral delivery.

The hERG parameter represents a critical safety indicator used to assess a compound’s potential to inhibit hERG potassium (K^+^) channels [29], an effect that may contribute to the development of long QT syndrome (LQTS), cardiac arrhythmias, and Torsade de Pointes. The obtained results indicate that all analyzed derivatives exhibit a moderate risk of this type of inhibitory activity, comparable to that observed for the reference compounds RM2 and RM3, while the risk associated with RM remains negligible.

The predicted Ames toxicity values suggest that none of the proposed compounds is expected to display mutagenic potential. In contrast, the reference molecules show a markedly different profile, with a substantial probability of mutagenicity ranging from 39% (RM3) to 83% (RM2).

The final evaluated parameter, CARC, reflecting carcinogenic potential, shows considerable variability among the analyzed compounds. Certain derivatives, such as 3b, 4b, and 4e, exhibit negligible carcinogenic risk, whereas others, including 2f and 4f, demonstrate an intermediate carcinogenic potential. Nevertheless, for all investigated compounds, the predicted carcinogenic probability remains lower than that observed for the reference molecules RM and RM2.

## 3. Materials and Methods

### 3.1. Ab-Initio Calculations

The geometric and electronic properties of the studied compounds were evaluated using Density Functional Theory (DFT) [36,37]. The structures of the investigated inhibitors were prepared using GaussView 6.0.16, and all quantum-chemical calculations were performed with the Gaussian 16 Rev. C.01 package [38], while the analysis of the frontier orbitals was carried out using the Avogadro 1.2.0 application [39]. Geometry optimizations and molecular properties were calculated at the B3LYP/6-31G (d,p) level of theory [40,41]. The energies of the HOMO and LUMO orbitals were used as source data for the estimation of several chemical reactivity descriptors, providing an opportunity to characterize a molecule’s chemical nature and reactivity. The analyses were based on the values of the energy gap (ΔE_GAP_) (2), absolute hardness (η) (3), absolute electronegativity (χ) (4), chemical potential (μ) (5), absolute softness (σ) (6), global softness (S) (7), global electrophilicity index (ω) (8), and the maximum amount of additional electronic charge (ΔN_MAX_) (9) [42,43,44].

(2) $${\Delta E}_{GAP}=E_{LUMO}-E_{HOMO}$$

(3) $$\eta =\frac{E_{LUMO}-E_{HOMO}}{2}$$

(4) $$\chi =-\frac{E_{HOMO}+E_{LUMO}}{2}$$

(5) $$\mu =-\chi =\frac{E_{HOMO}+E_{LUMO}}{2}$$

(6) $$\sigma =\frac{1}{\eta }$$

(7) $$S=\frac{1}{2\eta }$$

(8) $$\omega =\frac{\mu^{2}}{2\eta }$$

(9) $$\Delta N_{MAX}=-\frac{\mu }{\eta }$$

### 3.2. The Docking Procedure and In-Silico ADMET Analysis

The geometric structure of the enzyme cyclin-dependent kinase 2 (CDK2; PDB ID: 1E9H) was downloaded from the Protein Data Bank (PDB) [45]. During the docking procedure, two programs were used: initial preparation was performed using the AutoDockTools 1.5.6 package [46], while docking simulations were carried out using AutoDock Vina software [47]. During the initial preparatory steps, all non-polar hydrogen atoms were removed from all chemical structures, including the protein and the inhibitors; additionally, the number of rotatable bonds was defined for the considered ligands. The grid box dimensions of 16 × 14 × 22 Å were set, ensuring that the docking procedure fully covered the active-site space and enabled a reliable assessment of ligand affinity. The docking protocol was validated by re-docking the co-crystallized ligand into the CDK2 active site. The resulting geometry closely matched the experimental conformation, with the RMSD between the predicted pose and the original crystallographic binding mode well below 2.0 Å, confirming the reliability and accuracy of the selected docking parameters. For each inhibitor candidate, the docking procedure was repeated five times using different random seed values to ensure proper execution and to obtain representative and reproducible results. In all considered complexes, the predicted conformations were highly consistent and corresponded to the poses with the lowest predicted binding affinity, further supporting the validity of the docking approach. During the simulations, the exhaustiveness parameter was set to 20, which ensured an appropriate balance between precision and computational time.

The ADMET (Absorption, Distribution, Metabolism, Excretion, and Toxicity) properties of the considered molecules were evaluated using the ADMETlab 3.0 platform [48], which enables quantitative structure–activity relationship (QSAR) analysis based on two-dimensional molecular structures described by SMILES notation. Among the numerous available descriptors, those defining molecular properties (molecular weight, number of hydrogen bond donors (nHD), number of hydrogen bond acceptors (nHA), topological polar surface area (TPSA), logP, logD, and logS, absorption (Caco-2 permeability and human intestinal absorption (HIA)), distribution (blood–brain barrier penetration), and toxicity (hERG blockers, AMES toxicity, and carcinogenicity (CARC)) were selected to obtain a comparative characterization of the properties of the candidate molecules exhibiting the highest binding affinity to the CDK2 active site.

### 3.3. The Molecular Dynamics Simulations

During all molecular dynamics simulations, the protein component of the studied complexes (CDK2) was described using the ff14SB force field [49], while the ligand structures were parameterized using the General Amber Force Field (GAFF) [50]. Partial atomic charges for the inhibitors were derived using the Merz–Kollman scheme followed by the RESP fitting procedure at the HF/6-31G* level of theory [51]. Each molecular system considered during the molecular dynamics stage was neutralized with chloride ions and immersed in a periodic box of TIP3P water molecules. The initial phase consisted of energy minimization and gradual heating of the system to 300 K; temperature regulation was carried out using a Langevin thermostat [52]. After these preparatory steps, production molecular dynamics simulations were performed for 80 ns under periodic boundary conditions, with the SHAKE algorithm applied to constrain bonds involving hydrogen atoms.

The analysis of the studied complexes, including the estimation of structural stability, flexibility, and mobility of individual components of the tested systems, was carried out using the VMD package [53]. During the structural analysis of interactions within the active-site region, hydrogen bonds were defined according to the following criteria: a hydrogen–acceptor distance ≤ 3.0 Å, a donor–acceptor distance ≤ 3.5 Å, and a donor–hydrogen–acceptor angle greater than 90°. For selected systems, namely the apo CDK2 protein and its complexes with compounds 1, 2a, 3h, and 4e, extended molecular dynamics (MD) simulations of 160 ns were performed to investigate the potential effects of ligand binding on the active site. The simulations were analyzed in terms of root mean square fluctuations (RMSF) to characterize the flexibility and conformational dynamics of individual amino acid residues within the active site and its immediate surroundings. The molecular mechanics Poisson–Boltzmann surface area (MMPBSA) method [54] was employed to estimate the enthalpic contributions to binding affinity values characterizing the complexes formed by ligands with the CDK2 active site. The calculations were performed based on 100 randomly selected conformers of the analyzed systems from the last 60 ns of the molecular dynamics simulations. The molecular dynamics simulations were performed using the AMBER 14 package [55]. One-way analysis of variance (ANOVA) with the pairwise multiple-comparison Holm–Šidák method [56,57] was employed in the statistical analysis of mean values and standard deviations for RMSD and enthalpic contributions to binding affinity. All statistical tests were conducted with an overall significance level of α = 0.05.

## 4. Conclusions

The conducted studies, including quantum mechanical calculations, molecular docking, molecular dynamics simulations, and QSAR-based ADMET analysis, enabled a comprehensive evaluation of the investigated compounds as potential cyclin-dependent kinase 2 (CDK2) inhibitors. Structural diversity arising from electron-donating and electron-withdrawing substituents on the aromatic system allowed assessment of binding capabilities and identification of derivatives exhibiting the highest conformational stability within the enzyme active site. Docking results revealed relatively low variability in affinity across the compound set and emphasized the dominant role of the isatin core bearing a novel substituent that reorients the molecular scaffold and actively participates in intermolecular interactions.

The trifluoromethoxy substituent at the C6 position was identified as a key determinant of active-site orientation through hydrogen-bond formation with ASP145 and LYS33, producing a binding geometry distinct from previously studied C5-substituted isatin derivatives and altering interaction distances with GLU81 and LYS83. Hydrogen-bond and distance distribution analyses indicated deeper embedding of the hinge region within the hydrophobic pocket, particularly for representative derivatives **2a**, **3h**, and **4a**. Molecular dynamics simulations further confirmed favorable accommodation and conformational stability of selected compounds—**1**, **2a**, **3e**, **3h**, **3k**, **4f**, and **4i**—as evidenced by stable RMSD profiles and consistently low enthalpy values.

ADMET evaluation demonstrated compliance with fundamental drug-likeness criteria, with only moderate solubility limitations that remain below pharmacological exclusion thresholds and may be mitigated using binary solubility enhancers, as reported for analogous isatin derivatives. Additional descriptors indicated favorable absorption, distribution, and low toxicity or mutagenicity risk.

Overall, the strong affinity toward CDK2 and favorable physicochemical and pharmacokinetic properties—especially for the representative derivatives highlighted above—identify the proposed compounds as promising candidates for further investigation, including chemical synthesis and cellular evaluation.

## Figures and Tables

**Table 1:** Values of HOMO and LUMO energies, energy gap (ΔE_GAP_), absolute hardness (η), chemical potential (μ), absolute softness (σ), global softness (S), absolute electronegativity (χ), global electrophilicity index (ω), and maximum additional electronic charge (ΔN_MAX_).

Name	HOMO	LUMO	ΔE_GAP_	η	μ	σ	s	χ	ω	ΔN_MAX_
	[eV]	[eV]	[eV]	[eV]	[eV]	[eV^−1^]	[eV^−1^]	[eV]	[eV]	---
**1**	−6.49	−2.47	4.02	2.01	4.48	0.50	0.25	4.48	4.99	−2.23
**2a**	−6.48	−2.44	4.04	2.02	4.46	0.50	0.25	4.46	4.92	−2.21
**2c**	−6.51	−2.48	4.03	2.02	4.49	0.50	0.25	4.49	5.01	−2.23
**2f**	−5.95	−2.42	3.53	1.77	4.18	0.57	0.28	4.18	4.95	−2.37
**2i**	−6.45	−2.43	4.03	2.01	4.44	0.50	0.25	4.44	4.89	−2.20
**3a**	−6.48	−2.46	4.02	2.01	4.47	0.50	0.25	4.47	4.97	−2.22
**3b**	−6.52	−2.52	4.00	2.00	4.52	0.50	0.25	4.52	5.11	−2.26
**3c**	−6.51	−2.50	4.01	2.00	4.51	0.50	0.25	4.51	5.07	−2.25
**3d**	−6.52	−2.52	4.00	2.00	4.52	0.50	0.25	4.52	5.10	−2.26
**3e**	−6.52	−2.52	4.00	2.00	4.52	0.50	0.25	4.52	5.10	−2.26
**3h**	−6.54	−2.60	3.94	1.97	4.57	0.51	0.25	4.57	5.30	−2.32
**3k**	−6.22	−2.57	3.65	1.82	4.39	0.55	0.27	4.39	5.28	−2.41
**4a**	−6.17	−2.50	3.67	1.83	4.33	0.55	0.27	4.33	5.12	−2.36
**4b**	−6.22	−2.58	3.64	1.82	4.40	0.55	0.28	4.40	5.33	−2.42
**4c**	−6.19	−2.52	3.67	1.83	4.35	0.55	0.27	4.35	5.16	−2.37
**4d**	−6.20	−2.55	3.65	1.82	4.38	0.55	0.27	4.38	5.25	−2.40
**4e**	−6.20	−2.55	3.65	1.83	4.38	0.55	0.27	4.38	5.24	−2.40
**4f**	−6.01	−2.38	3.64	1.82	4.19	0.55	0.28	4.19	4.84	−2.31
**4i**	−6.11	−2.47	3.64	1.82	4.29	0.55	0.27	4.29	5.05	−2.36

**Table 2:** The values of binding affinity and inhibition constants (IC) of 6-Trifluoromethoxyisatin-based benzoylhydrazides towards CDK2 active site. The value for native molecule (1) is −9.80 [kcal/mol].

Chemical Group	Name	Binding Affinity [kcal/mol]	IC [nM]	Name	Binding Affinity [kcal/mol]	IC [nM]	Name	Binding Affinity [kcal/mol]	IC [nM]
**–CH_3_**	2a	−9.90	55.36	3a	−10.08	40.86	4a	−10.00	46.76
**–CF_3_**	2b	−9.86	59.23	3b	−10.10	39.50	4b	−10.00	46.76
**–F**	2c	−10.00	46.76	3c	−10.04	43.71	4c	−9.90	55.36
**–Br**	2d	−9.60	91.86	3d	−9.90	55.36	4d	−9.90	55.36
**–Cl**	2e	−9.80	65.54	3e	−9.90	55.36	4e	−9.90	55.36
**–NH_2_**	2f	−9.90	55.36	3f	−9.80	65.54	4f	−9.90	55.36
**–N(CH_3_)_2_**	2g	−8.28	852.47	3g	−9.80	65.54	4g	−9.78	67.79
**–NO_2_**	2h	−9.58	95.01	3h	−9.90	55.36	4h	−9.80	65.54
**–OH**	2i	−9.90	55.36	3i	−9.80	65.54	4i	−9.90	55.36
**–OCH_3_**	2j	−9.30	152.41	3j	−9.80	65.54	4j	−9.70	77.59
**–OCF_3_**	2k	−8.68	433.99	3k	−9.90	55.36	4k	−9.78	67.79

**Table 3:** Values of hydrogen bond lengths and distances between aromatic systems involved in the stabilization of complexes obtained at the docking stage.

Name	Distances [Å]						
	GLU 81	LEU 83 H	LEU 83 O	PHE 80	PHE 82	ASP 145	LYS 33
1	2.63	1.83	2.32	4.07	3.90	1.78	3.01
2a	2.36	1.73	2.59	3.74	3.70	1.69	2.47
2c	2.61	1.81	2.27	4.06	3.69	1.75	2.46
2f	2.63	1.81	2.25	4.09	3.69	1.78	2.47
2i	2.61	1.81	2.28	4.05	3.71	1.76	2.43
3a	2.66	1.89	2.44	4.06	4.14	1.75	2.43
3b	2.59	1.88	2.52	3.97	4.23	2.23	2.52
3c	2.63	1.83	2.34	4.07	3.95	1.78	2.39
3d	2.63	1.82	2.26	4.08	3.73	1.77	2.43
3e	2.67	1.90	2.44	4.06	4.14	1.76	2.42
3h	2.62	1.82	2.29	4.06	3.80	1.77	2.42
3k	2.66	1.83	2.27	4.11	3.88	1.78	2.39
4a	2.63	1.83	2.32	4.06	3.91	1.77	2.38
4b	2.67	1.87	2.36	4.10	4.03	1.77	2.40
4c	2.64	1.82	2.28	4.08	3.83	1.77	2.40
4d	2.63	1.84	2.36	4.06	4.01	1.78	2.41
4e	2.64	1.82	2.30	4.08	3.89	1.78	2.38
4f	2.63	1.81	2.26	4.07	3.74	1.78	2.42
4i	2.63	1.81	2.25	4.08	3.72	1.78	2.43
RM	2.41	2.04	----	3.70	5.17	----	----

**Table 4:** Averaged RMSD values for ligands and the CDK2 protein for conformers from the 80 ns and the final 60 ns of the molecular dynamics simulation.

Name	Ligand				CDK2			
	80 ns		Last 60 ns		80 ns		Last 60 ns	
	RMSD	SD	RMSD	SD	RMSD	SD	RMSD	SD
**1**	**0.83**	**0.17**	**0.86**	**0.16**	3.56	0.65	3.84	0.39
**2a**	**0.98**	**0.16**	**1.00**	**0.15**	3.06	0.42	3.21	0.32
**2c**	1.01	0.38	1.09	0.39	2.91	0.33	3.01	0.20
**2f**	0.94	0.19	0.98	0.18	2.67	0.32	2.76	0.20
**2i**	**0.85**	**0.19**	**0.86**	**0.18**	2.84	0.38	2.96	0.30
**3a**	1.32	0.27	1.41	0.11	**2.59**	**0.29**	**2.63**	**0.25**
**3b**	1.35	0.43	1.49	0.39	2.46	0.23	2.54	0.17
**3c**	1.16	0.41	1.21	0.45	2.70	0.27	2.74	0.20
**3d**	**0.78**	**0.20**	**0.80**	**0.19**	3.16	0.58	3.43	0.31
**3e**	**1.02**	**0.22**	**0.99**	**0.19**	2.98	0.41	3.13	0.33
**3h**	1.40	0.26	1.44	0.16	2.66	0.27	2.76	0.15
**3k**	**1.15**	**0.21**	**1.16**	**0.19**	2.71	0.33	2.82	0.22
**4a**	1.26	0.28	1.38	0.13	2.75	0.37	2.89	0.25
**4b**	1.31	0.21	1.38	0.16	3.08	0.54	3.30	0.35
**4c**	**0.83**	**0.23**	**0.83**	**0.24**	2.70	0.37	2.76	0.35
**4d**	0.83	0.24	0.79	0.21	3.13	0.41	3.25	0.26
**4e**	1.05	0.43	1.13	0.46	2.89	0.36	3.04	0.24
**4f**	1.09	0.18	1.13	0.16	2.61	0.33	2.74	0.25
**4i**	**0.86**	**0.14**	**0.85**	**0.13**	2.91	0.29	3.01	0.19

**Table 5:** The cumulative analysis of the length of the interactions identified in CDK2 complexes with selected 6-Trifluoromethoxyisatin-based benzoylhydrazides. The distances presented in the table represent middle values of intervals with a width of 0.2 Å.

Interactions	Population %								
	Σ	1.6 Å	1.8 Å	2 Å	2.2 Å	2.4 Å	2.6 Å	2.8 Å	3 Å
1									
Ligand (**H1**) … (**O**) GLU 81	100.0	1.6	41.0	44.5	11.2	1.4	0.3	0.1	0.0
Ligand (**O1**) … (**HN**) LEU 83	99.9	0.8	25.9	44.9	20.4	5.6	2.1	0.3	0.0
Ligand (**H5**) … (**O**) LEU 83	38.3	0.0	0.1	1.3	3.3	4.4	6.6	9.5	13.0
Ligand (**F**) … (**HN**) LYS 33	44.7	0.0	1.3	6.3	8.1	9.0	6.4	6.9	6.7
Ligand (**F**) … (**H**) ASP145	85.9	0.0	3.4	16.0	23.1	20.1	12.3	6.9	4.2
2a									
Ligand (**H1**) … (**O**) GLU 81	100.0	4.3	58.8	30.3	5.5	0.9	0.1	0.1	0.0
Ligand (**O2**) … (**HN**) LEU 83	100.0	0.5	26.8	46.0	19.6	5.6	1.0	0.4	0.2
Ligand (**H5**) … (**O**) LEU 83	7.3	0.0	0.1	0.1	0.8	1.1	0.6	1.8	2.9
3e									
Ligand (**H1**) … (**O**) GLU 81	100.0	2.1	47.4	39.8	9.6	0.9	0.3	0.0	0.0
Ligand (**O1**) … (**HN**) LEU 83	99.8	0.8	38.9	44.8	12.9	2.1	0.4	0.1	0.0
Ligand (**H5**) … (**O**) LEU 83	80.2	0.1	7.1	24.1	17.2	11.5	7.8	5.9	6.4
Ligand (**F**) … (**HN**) LYS 33	45.8	0.0	0.0	1.2	3.7	7.3	11.0	12.1	10.6
Ligand (**F**) … (**H**) ASP145	39.3	0.0	0.1	1.7	4.4	5.8	7.3	10.0	9.9
3h									
Ligand (**H1**) … (**O**) GLU 81	100.0	2.6	47.6	41.3	7.4	1.0	0.0	0.0	0.0
Ligand (**O1**) … (**HN**) LEU 83	100.0	1.3	34.7	46.4	14.3	2.9	0.4	0.1	0.0
Ligand (**H5**) … (**O**) LEU 83	84.1	0.0	0.0	0.0	1.8	20.1	28.6	20.2	13.5
Ligand (**F**) … (**HN**) LYS 33	34.1	0.0	0.1	0.3	1.1	3.8	7.8	10.3	10.8
Ligand (**F**) … (**H**) ASP145	95.2	0.3	10.1	26.6	25.0	15.8	9.8	4.9	2.8
3k									
Ligand (**H1**) … (**O**) GLU 81	100.0	1.3	47.9	41.6	8.3	0.9	0.1	0.0	0.0
Ligand (**O1**) … (**HN**) LEU 83	100.0	1.1	41.3	45.0	10.8	1.6	0.4	0.0	0.0
Ligand (**H5**) … (**O**) LEU 83	78.3	0.0	2.8	13.6	14.4	14.2	11.6	12.2	9.4
Ligand (**F**) … (**HN**) LYS 33	45.9	0.0	0.1	0.7	2.9	6.9	10.6	12.5	12.3
Ligand (**F**) … (**H**) ASP145	83.7	0.0	2.4	11.7	16.8	16.5	13.8	13.8	8.8
4e									
Ligand (**H1**) … (**O**) GLU 81	100.0	1.1	39.3	46.5	11.1	1.4	0.5	0.1	0.1
Ligand (**O1**) … (**HN**) LEU 83	100.0	0.9	33.6	45.4	15.4	4.0	0.6	0.1	0.0
Ligand (**H5**) … (**O**) LEU 83	42.0	0.0	0.7	5.5	5.5	6.3	7.1	7.8	9.2
Ligand (**F**) … (**HN**) LYS 33	32.7	0.0	0.0	0.5	1.9	4.1	7.3	8.6	10.3
Ligand (**F**) … (**H**) ASP145	61.8	0.0	3.4	8.7	9.4	10.8	9.5	9.5	10.6
Ligand (**O1**) … (**H5**) Ligand	96.4	0.0	0.0	3.6	20.6	33.2	20.8	12.6	5.6
4f									
Ligand (**H1**) … (**O**) GLU 81	100.0	1.7	42.6	43.9	10.1	1.3	0.3	0.1	0.0
Ligand (**O1**) … (**HN**) LEU 83	100.0	0.9	31.1	45.9	17.0	4.1	0.7	0.2	0.1
Ligand (**H5**) … (**O**) LEU 83	41.1	0.0	0.6	4.2	6.3	6.5	5.9	8.4	9.2
Ligand (**F**) … (**HN**) LYS 33	24.6	0.0	0.0	0.4	0.7	2.4	5.2	7.0	8.8
Ligand (**F**) … (**H**) ASP145	87.4	0.0	4.3	18.4	23.3	18.8	11.2	6.4	5.1
4i									
Ligand (**H1**) … (**O**) GLU 81	100.0	1.3	44.8	44.0	8.9	0.9	0.2	0.0	0.0
Ligand (**O1**) … (**HN**) LEU 83	100.0	1.3	37.6	45.0	12.5	3.0	0.5	0.1	0.1
Ligand (**H5**) … (**O**) LEU 83	65.4	0.3	3.6	10.3	12.0	9.8	9.0	9.3	11.2
Ligand (**F**) … (**HN**) LYS 33	37.1	0.0	0.0	0.8	1.9	5.4	8.0	10.4	10.7
Ligand (**F**) … (**H**) ASP145	48.4	0.0	0.4	2.8	5.9	9.6	9.4	9.8	10.6
RM									
Ligand (**H1**) … (**O**) GLU 81	99.94	1.31	39.44	44.25	12.94	1.75	0.25	0.00	0.00
Ligand (**O1**) … (**HN**) LEU 83	98.94	0.13	5.69	23.81	27.25	19.75	12.06	7.13	3.13

**Table 6:** ADMET parameters evaluated for considered molecules and reference system using ADMETlab 3.0.

Name	MW ^a^	nHD ^b^	nHA ^c^	TPSA ^d^	LogP ^e^	LogD ^f^	LogS ^g^	BBB ^h^	Caco-2 ^i^	HIA ^j^	hERG ^k^	AMES ^l^	CARC ^m^
**1**	349.07	2	6	79.8	3.35	3.30	−4.75	6.5 × 10^−6^	−4.87	0.0004	0.52	0.09	0.35
**2a**	363.08	2	6	79.8	3.36	3.34	−4.72	4.3 × 10^−6^	−4.85	0.0002	0.51	0.10	0.54
**2c**	367.06	2	6	79.8	3.26	3.29	−4.73	6.9 × 10^−6^	−4.91	0.0003	0.59	0.15	0.49
**2f**	364.08	4	7	106	3.23	3.24	−4.62	2.2 × 10^−6^	−5.12	0.0005	0.48	0.23	0.60
**2i**	365.06	3	7	100	3.66	3.27	−5.22	1.5 × 10^−6^	−5.07	0.0013	0.34	0.11	0.37
**3a**	363.08	2	6	79.8	3.58	3.44	−4.94	1.4 × 10^−6^	−4.87	0.0001	0.53	0.07	0.36
**3b**	417.05	2	6	79.8	3.89	3.58	−5.11	1.7 × 10^−5^	−4.95	0.0002	0.62	0.02	0.12
**3c**	367.06	2	6	79.8	3.49	3.34	−4.91	1.2 × 10^−6^	−4.84	0.0001	0.57	0.09	0.38
**3d**	426.98	2	6	79.8	3.75	3.53	−4.97	7.0 × 10^−6^	−4.96	0.0003	0.54	0.04	0.34
**3e**	383.03	2	6	79.8	3.65	3.50	−4.96	3.0 × 10^−6^	−4.83	0.0001	0.65	0.05	0.31
**3h**	394.05	2	9	123	3.31	3.31	−4.99	2.7 × 10^−8^	−4.99	0.0001	0.53	0.34	0.44
**3k**	433.05	2	7	89	3.59	3.24	−4.59	2.1 × 10^−6^	−4.93	0.0003	0.86	0.01	0.18
**4a**	363.08	2	6	79.8	3.57	3.45	−4.95	8.6 × 10^−6^	−4.84	0.0006	0.54	0.07	0.36
**4b**	417.05	2	6	79.8	3.75	3.40	−4.85	2.7 × 10^−5^	−4.92	0.0008	0.66	0.02	0.11
**4c**	367.06	2	6	79.8	3.45	3.26	−4.87	7.0 × 10^−5^	−4.82	0.0003	0.56	0.09	0.45
**4d**	426.98	2	6	79.8	3.71	3.44	−4.83	2.8 × 10^−4^	−4.97	0.0002	0.58	0.03	0.34
**4e**	383.03	2	6	79.8	3.56	3.37	−4.76	1.2 × 10^−4^	−4.84	0.0001	0.68	0.04	0.30
**4f**	364.08	4	7	106	2.97	2.82	−4.28	1.6 × 10^−6^	−5.16	0.0001	0.40	0.34	0.62
**4i**	365.06	3	7	100	3.32	3.16	−5.06	2.7 × 10^−7^	−5.09	0.0041	0.50	0.07	0.37
**RM**	317.01	1	4	53.8	2.89	2.98	−4.50	6.5 × 10^−7^	−4.67	0.0002	0.13	0.57	0.70
**RM2**	354.22	3	7	87.9	4.00	3.66	−4.85	0.125	−5.29	0.0001	0.50	0.90	0.83
**RM3**	396.23	2	8	92.6	2.64	2.60	−4.21	0.407	−5.58	0.0013	0.49	0.39	0.43

^a^ Molecular weight (<500 u); ^b^ number of hydrogen bond donors (nHD ≤ 5); ^c^ number of hydrogen bond acceptors (nHA ≤ 10); ^d^ Topological Polar Surface Area (TPSA ≤ 140); ^e^ log of the octanol/water partition coefficient (optimal 0~3; <5); ^f^ logP at physiological pH 7.4 (Optimal: 1~3; <5); ^g^ log of the aqueous solubility (optimal: −4~0.5); ^h^ Blood–Brain Barrier Penetration; the value is the probability of being BBB+; ^i^ Caco-2 permeability (optimal: higher than −5.15 log cm/s); ^j^ Human Intestinal Absorption, Category 1: HIA + (HIA < 30%); Category 0: HIA-(HIA < 30%); the output value is the probability of being HIA+; ^k^ the output value is the probability of being active blocker of hERG K+ channels; ^l^ the output value is the probability of exhibiting mutagenic properties; ^m^ the output value is the probability of exhibiting carcinogenic properties.

## Data Availability

All data are placed in the article and Supplementary Materials.

## Supplementary Materials

The following supporting information can be downloaded at: https://www.mdpi.com/article/10.3390/ijms27041802/s1.

## Abbreviations

The following abbreviations are used in this manuscript:

CDK2	Cyclin-Dependent Kinase 2
ADMET	Absorption, Distribution, Metabolism, Excretion, and Toxicity
MMPBSA	The Molecular Mechanics Poisson–Boltzmann Surface Area
QSAR	Quantitative Structure–Activity Relationship
GAFF	General Amber Force Field
PDB	Protein Data Bank
RMSD	Root Mean Square Deviation
RMSF	Root Mean Square Fluctuation
ANOVA	One-way analysis of variance
MW	Molecular weight
nHD	Number of hydrogen bond donors
nHA	Number of hydrogen bond acceptors
TPSA	Topological Polar Surface Area
LogP	Log of the octanol/water partition coefficient
LogD	logP at physiological pH 7.4
LogS	Log of the aqueous solubility
BBB	Brain Barrier Penetration
HIA	Human Intestinal Absorption
RM	Reference Molecule

## References

[1] Boire A., Burke K., Cox T.R., Guise T., Jamal-Hanjani M., Janowitz T., Kaplan R., Lee R., Swanton C., Vander Heiden M.G. (2024). Why do patients with cancer die?. Nat. Rev. Cancer.

[2] Hjartåker A., Weiderpass E., Bray F. (2017). Cancer Mortality. International Encyclopedia of Public Health.

[3] Morgan D.O. (1997). Cyclin-Dependent Kinases: Engines, Clocks, and Microprocessors. Annu. Rev. Cell Dev. Biol..

[4] Malumbres M., Barbacid M. (2009). Cell cycle, CDKs and cancer: A changing paradigm. Nat. Rev. Cancer.

[5] Besson A., Dowdy S.F., Roberts J.M. (2008). CDK Inhibitors: Cell Cycle Regulators and Beyond. Dev. Cell.

[6] Ghafouri-Fard S., Khoshbakht T., Hussen B.M., Dong P., Gassler N., Taheri M., Baniahmad A., Dilmaghani N.A. (2022). A review on the role of cyclin dependent kinases in cancers. Cancer Cell Int..

[7] Ferraz de Paiva R.E., Vieira E.G., Rodrigues da Silva D., Wegermann C.A., Costa Ferreira A.M. (2021). Anticancer Compounds Based on Isatin-Derivatives: Strategies to Ameliorate Selectivity and Efficiency. Front. Mol. Biosci..

[8] Lashen A., Alqahtani S., Shoqafi A., Algethami M., Jeyapalan J.N., Mongan N.P., Rakha E.A., Madhusudan S. (2024). Clinicopathological Significance of Cyclin-Dependent Kinase 2 (CDK2) in Ductal Carcinoma In Situ and Early-Stage Invasive Breast Cancers. Int. J. Mol. Sci..

[9] Sabnis R.W. (2020). Novel CDK2 Inhibitors for Treating Cancer. ACS Med. Chem. Lett..

[10] Chenette E.J. (2010). A key role for CDK2. Nat. Rev. Cancer.

[11] Ghosh S., Ramarao T.A., Samanta P.K., Jha A., Satpati P., Sen A. (2021). Triazole based isatin derivatives as potential inhibitor of key cancer promoting kinases- insight from electronic structure, docking and molecular dynamics simulations. J. Mol. Graph. Model..

[12] Shin E.-K., Kim J.-K. (2012). Indirubin derivative E804 inhibits angiogenesis. BMC Cancer.

[13] Bramson H.N., Holmes W.D., Hunter R.N., Lackey K.E., Lovejoy B., Luzzio M.J., Montana V., Rocque W.J., Rusnak D., Shewchuk L. (2001). Oxindole-based inhibitors of cyclin-dependent kinase 2 (CDK2): Design, synthesis, enzymatic activities, and X-ray crystallographic analysis. J. Med. Chem..

[14] Sabt A., Eldehna W.M., Al-Warhi T., Alotaibi O.J., Elaasser M.M., Suliman H., Abdel-Aziz H.A. (2020). Discovery of 3,6-disubstituted pyridazines as a novel class of anticancer agents targeting cyclin-dependent kinase 2: Synthesis, biological evaluation and in silico insights. J. Enzyme Inhib. Med. Chem..

[15] Czeleń P., Skotnicka A., Szefler B., Kabatc-Borcz J., Sutkowy P. (2025). Design and Synthesis of New 5-Methylisatin Derivatives as Potential CDK2 Inhibitors. Int. J. Mol. Sci..

[16] Czeleń P., Szefler B. (2021). The Oxindole Derivatives, New Promising GSK-3β Inhibitors as One of the Potential Treatments for Alzheimer’s Disease—A Molecular Dynamics Approach. Biology.

[17] Czeleń P., Skotnicka A., Szefler B. (2022). Designing and Synthesis of New Isatin Derivatives as Potential CDK2 Inhibitors. Int. J. Mol. Sci..

[18] Czeleń P. (2019). Investigation of the Inhibition Potential of New Oxindole Derivatives and Assessment of Their Usefulness for Targeted Therapy. Symmetry.

[19] Ansari M., Ghandadi M., Emami S. (2025). An overview of isatin-derived CDK2 inhibitors in developing anticancer agents. Eur. J. Med. Chem..

[20] Al-Sanea M.M., Obaidullah A.J., Shaker M.E., Chilingaryan G., Alanazi M.M., Alsaif N.A., Alkahtani H.M., Alsubaie S.A., Abdelgawad M.A. (2021). A New CDK2 Inhibitor with 3-Hydrazonoindolin-2-One Scaffold Endowed with Anti-Breast Cancer Activity: Design, Synthesis, Biological Evaluation, and In Silico Insights. Molecules.

[21] Alanazi M.M., Alanazi A.S. (2023). Novel 7-Deazapurine Incorporating Isatin Hybrid Compounds as Protein Kinase Inhibitors: Design, Synthesis, In Silico Studies, and Antiproliferative Evaluation. Molecules.

[22] Alanazi A.S., Mirgany T.O., Alsfouk A.A., Alsaif N.A., Alanazi M.M. (2023). Antiproliferative Activity, Multikinase Inhibition, Apoptosis- Inducing Effects and Molecular Docking of Novel Isatin–Purine Hybrids. Medicina.

[23] Eldehna W.M., Al-Rashood S.T., Al-Warhi T., Eskandrani R.O., Alharbi A., El Kerdawy A.M. (2021). Novel oxindole/benzofuran hybrids as potential dual CDK2/GSK-3β inhibitors targeting breast cancer: Design, synthesis, biological evaluation, and in silico studies. J. Enzyme Inhib. Med. Chem..

[24] Eldehna W.M., El Hassab M.A., Abo-Ashour M.F., Al-Warhi T., Elaasser M.M., Safwat N.A., Suliman H., Ahmed M.F., Al-Rashood S.T., Abdel-Aziz H.A. (2021). Development of isatin-thiazolo[3,2-a]benzimidazole hybrids as novel CDK2 inhibitors with potent in vitro apoptotic anti-proliferative activity: Synthesis, biological and molecular dynamics investigations. Bioorg. Chem..

[25] Czeleń P., Jeliński T., Skotnicka A., Szefler B., Szupryczyński K. (2023). ADMET and Solubility Analysis of New 5-Nitroisatine-Based Inhibitors of CDK2 Enzymes. Biomedicines.

[26] Xiong G., Wu Z., Yi J., Fu L., Yang Z., Hsieh C., Yin M., Zeng X., Wu C., Lu A. (2021). ADMETlab 2.0: An integrated online platform for accurate and comprehensive predictions of ADMET properties. Nucleic Acids Res..

[27] Daina A., Michielin O., Zoete V. (2017). SwissADME: A free web tool to evaluate pharmacokinetics, drug-likeness and medicinal chemistry friendliness of small molecules. Sci. Rep..

[28] Lei T., Li Y., Song Y., Li D., Sun H., Hou T. (2016). ADMET evaluation in drug discovery: 15. Accurate prediction of rat oral acute toxicity using relevance vector machine and consensus modeling. J. Cheminform..

[29] Wang S., Sun H., Liu H., Li D., Li Y., Hou T. (2016). ADMET Evaluation in Drug Discovery. 16. Predicting hERG Blockers by Combining Multiple Pharmacophores and Machine Learning Approaches. Mol. Pharm..

[30] Cheng W., Yang Z., Wang S., Li Y., Wei H., Tian X., Kan Q. (2019). Recent development of CDK inhibitors: An overview of CDK/inhibitor co-crystal structures. Eur. J. Med. Chem..

[31] Chohan T.A., Qayyum A., Rehman K., Tariq M., Akash M.S.H. (2018). An insight into the emerging role of cyclin-dependent kinase inhibitors as potential therapeutic agents for the treatment of advanced cancers. Biomed. Pharmacother..

[32] Gupta P., Narayanan S., Yang D.-H. (2019). CDK Inhibitors as Sensitizing Agents for Cancer Chemotherapy. Protein Kinase Inhibitors as Sensitizing Agents for Chemotherapy.

[33] Lipinski C.A., Lombardo F., Dominy B.W., Feeney P.J. (2001). Experimental and computational approaches to estimate solubility and permeability in drug discovery and development settings. Adv. Drug Deliv. Rev..

[34] Prasanna S., Doerksen R.J. (2009). Topological Polar Surface Area: A Useful Descriptor in 2D-QSAR. Curr. Med. Chem..

[35] Wang N.-N., Dong J., Deng Y.-H., Zhu M.-F., Wen M., Yao Z.-J., Lu A.-P., Wang J.-B., Cao D.-S. (2016). ADME Properties Evaluation in Drug Discovery: Prediction of Caco-2 Cell Permeability Using a Combination of NSGA-II and Boosting. J. Chem. Inf. Model..

[36] Kohn W., Sham L.J. (1965). Self-Consistent Equations Including Exchange and Correlation Effects. Phys. Rev..

[37] Hohenberg P., Kohn W. (1964). Inhomogeneous Electron Gas. Phys. Rev..

[38] Frisch M.J., Trucks G.W., Schlegel H.B., Scuseria G.E., Robb M.A., Cheeseman J.R., Scalmani G., Barone V., Petersson G.A., Nakatsuji H. (2016). Gaussian 16.

[39] Hanwell M.D., Curtis D.E., Lonie D.C., Vandermeerschd T., Zurek E., Hutchison G.R. (2012). Avogadro: An advanced semantic chemical editor, visualization, and analysis platform. J. Cheminform..

[40] Becke A.D. (1993). Density-functional thermochemistry. III. The role of exact exchange. J. Chem. Phys..

[41] Ditchfield R., Hehre W.J., Pople J.A. (1971). Self-Consistent Molecular-Orbital Methods. IX. An Extended Gaussian-Type Basis for Molecular-Orbital Studies of Organic Molecules. J. Chem. Phys..

[42] Parr R.G., Pearson R.G. (1983). Absolute hardness: Companion parameter to absolute electronegativity. J. Am. Chem. Soc..

[43] Parr R.G., Szentpály L.V., Liu S. (1999). Electrophilicity Index. J. Am. Chem. Soc..

[44] Koopmans T. (1934). Über die Zuordnung von Wellenfunktionen und Eigenwerten zu den Einzelnen Elektronen Eines Atoms. Physica.

[45] Davies T.G., Tunnah P., Meijer L., Marko D., Eisenbrand G., Endicott J.A., Noble M.E. (2001). Inhibitor binding to active and inactive CDK2: The crystal structure of CDK2-cyclin A/indirubin-5-sulphonate. Structure.

[46] Bartashevich E.V., Potemkin V.A., Grishina M.A., Belik A.V. (2002). A Method for Multiconformational Modeling of the Three-Dimensional Shape of a Molecule. J. Struct. Chem..

[47] Trott O., Olson A.J. (2009). AutoDock Vina: Improving the speed and accuracy of docking with a new scoring function, efficient optimization, and multithreading. J. Comput. Chem..

[48] Fu L., Shi S., Yi J., Wang N., He Y., Wu Z., Peng J., Deng Y., Wang W., Wu C. (2024). ADMETlab 3.0: An updated comprehensive online ADMET prediction platform enhanced with broader coverage, improved performance, API functionality and decision support. Nucleic Acids Res..

[49] Maier J.A., Martinez C., Kasavajhala K., Wickstrom L., Hauser K.E., Simmerling C. (2015). ff14SB: Improving the Accuracy of Protein Side Chain and Backbone Parameters from ff99SB. J. Chem. Theory Comput..

[50] Wang J., Wolf R.M., Caldwell J.W., Kollman P.A., Case D.A. (2004). Development and testing of a general amber force field. J. Comput. Chem..

[51] Bayly C.I., Cieplak P., Cornell W., Kollman P.A. (1993). A well-behaved electrostatic potential based method using charge restraints for deriving atomic charges: The RESP model. J. Phys. Chem..

[52] Adelman S.A. (1976). Generalized Langevin equation approach for atom/solid-surface scattering: General formulation for classical scattering off harmonic solids. J. Chem. Phys..

[53] Humphrey W., Dalke A., Schulten K. (1996). VMD: Visual molecular dynamics. J. Mol. Graph..

[54] Miller B.R., McGee T.D., Swails J.M., Homeyer N., Gohlke H., Roitberg A.E. (2012). MMPBSA.py: An Efficient Program for End-State Free Energy Calculations. J. Chem. Theory Comput..

[55] Case D.A., Babin V., Berryman J.T., Betz R.M., Cai Q., Cerutti D.S., Cheatham T.E., Darden T.A., Duke R.E., Gohlke H. (2014). AMBER 14.

[56] Kelava A., Moosbrugger H., Dimitruk P., Schermelleh-Engel K. (2008). Methodology: European Journal of Research Methods for the Behavioral and Social Sciences. Methodology.

[57] Stoline M.R. (1981). The Status of Multiple Comparisons: Simultaneous Estimation of All Pairwise Comparisons in One-Way ANOVA Designs. Am. Stat..

