# Supplementary material for: Design and Evaluation of New 6-Trifluoromethoxy-Isatin Derivatives as Potential CDK2 Inhibitors
Source: Int J Mol Sci. 2026 Feb 13;27(4):1802. doi: 10.3390/ijms27041802 (PMC12940416; doi:10.3390/ijms27041802)
Supplement: Supplementary file 1 [file ijms-27-01802-s001.zip › ijms-4100111-supplementary.pdf]

Article

Design and Evaluation of New 6-Trifluoromethoxy-Isatin Derivatives as Potential CDK2 Inhibitors

Przemysław Czeleń * and Beata Szefler

Department of Physical Chemistry, Faculty of Pharmacy, Collegium Medicum, Nicolaus Copernicus University, Kurpinskiego 5, 85-096 Bydgoszcz, Poland; beatas@cm.umk.pl
* Correspondence: przemekcz@cm.umk.pl

Table S1. Summary of designations, chemical names, and SMILES codes for the derivative structures investigated in the study

Label	Chemical name / SMILES
**1**	*N*'-[6-Trifluoromethoxy-2-oxo-1,2-dihydro-3*H*-indol-3-ylidene]benzohydrazide<br>c12ccc(OC(F)(F)F)cc2[NH2+]C(=O)/C/1=N\NC(=O)c1ccccc1
**2a**	*N*'-[6-Trifluoromethoxy-2-oxo-1,2-dihydro-3*H*-indol-3-ylidene]-2-methylbenzohydrazide<br>c12ccc(cc2[NH2+]C(=O)/C/1=N\NC(=O)c1ccccc1C)OC(F)(F)F
**2c**	*N*'-[6-Trifluoromethoxy-2-oxo-1,2-dihydro-3*H*-indol-3-ylidene]-2-fluorobenzohydrazide<br>c12ccc(cc2[NH2+]C(=O)/C/1=N\NC(=O)c1ccccc1F)OC(F)(F)F
**2f**	*N*'-[6-Trifluoromethoxy-2-oxo-1,2-dihydro-3*H*-indol-3-ylidene]-2-aminobenzohydrazide<br>c12ccc(cc2[NH2+]C(=O)/C/1=N\NC(=O)c1ccccc1N)OC(F)(F)F
**2i**	*N*'-[6-Trifluoromethoxy-2-oxo-1,2-dihydro-3*H*-indol-3-ylidene]-2-hydroxybenzohydrazide<br>c12ccc(cc2[NH2+]C(=O)/C/1=N\NC(=O)c1ccccc1O)OC(F)(F)F
**3a**	*N*'-[6-Trifluoromethoxy-2-oxo-1,2-dihydro-3*H*-indol-3-ylidene]-3-methylbenzohydrazide<br>c12ccc(cc2[NH2+]C(=O)/C/1=N\NC(=O)c1cccc(c1)C)OC(F)(F)F
**3b**	*N*'-[6-Trifluoromethoxy-2-oxo-1,2-dihydro-3*H*-indol-3-ylidene]-3-(trifluoromethyl)benzohydrazide<br>c12ccc(cc2[NH2+]C(=O)/C/1=N\NC(=O)c1cccc(c1)C(F)(F)F)OC(F)(F)F
**3c**	*N*'-[6-Trifluoromethoxy-2-oxo-1,2-dihydro-3*H*-indol-3-ylidene]-3-fluorobenzohydrazide<br>c12ccc(cc2[NH2+]C(=O)/C/1=N\NC(=O)c1cccc(c1)F)OC(F)(F)F
**3d**	*N*'-[6-Trifluoromethoxy-2-oxo-1,2-dihydro-3*H*-indol-3-ylidene]-3-bromobenzohydrazide<br>c12ccc(cc2[NH2+]C(=O)/C/1=N\NC(=O)c1cccc(c1)Br)OC(F)(F)F
**3e**	*N*'-[6-Trifluoromethoxy-2-oxo-1,2-dihydro-3*H*-indol-3-ylidene]-3-chlorobenzohydrazide<br>c12ccc(cc2[NH2+]C(=O)/C/1=N\NC(=O)c1cccc(c1)Cl)OC(F)(F)F
**3h**	*N*'-[6-Trifluoromethoxy-2-oxo-1,2-dihydro-3*H*-indol-3-ylidene]-3-(trifluoromethoxy)benzohydrazide<br>O=C1Nc2cc(OC(F)(F)F)ccc2/C1=N/NC(=O)c1cccc([N+](=O)[O-])c1
**3k**	*N*'-[6-Trifluoromethoxy-2-oxo-1,2-dihydro-3*H*-indol-3-ylidene]-4-bromobenzohydrazide<br>c12ccc(cc2[NH2+]C(=O)/C/1=N\NC(=O)c1cccc(c1)OC(F)(F)F)OC(F)(F)F

**4a**	*N*'-[6-Trifluoromethoxy-2-oxo-1,2-dihydro-3*H*-indol-3-ylidene]-4-methylbenzohydrazide<br>c12ccc(cc2[NH2+]C(=O)/C/1=N\NC(=O)c1ccc(cc1)C)OC(F)(F)F
**4b**	*N*'-[6-Trifluoromethoxy-2-oxo-1,2-dihydro-3*H*-indol-3-ylidene]-4-(trifluoromethyl)benzohydrazide<br>c12ccc(cc2[NH2+]C(=O)/C/1=N\NC(=O)c1ccc(C(F)(F)F)cc1)OC(F)(F)F
**4c**	*N*'-[6-Trifluoromethoxy-2-oxo-1,2-dihydro-3*H*-indol-3-ylidene]-4-fluorobenzohydrazide<br>c12ccc(cc2[NH2+]C(=O)/C/1=N\NC(=O)c1ccc(cc1)F)OC(F)(F)F
**4d**	*N*'-[6-Trifluoromethoxy-2-oxo-1,2-dihydro-3*H*-indol-3-ylidene]-4-bromobenzohydrazide<br>c12ccc(cc2[NH2+]C(=O)/C/1=N\NC(=O)c1ccc(cc1)Br)OC(F)(F)F
**4e**	*N*'-[6-Trifluoromethoxy-2-oxo-1,2-dihydro-3*H*-indol-3-ylidene]-4-chlorobenzohydrazide<br>c12ccc(cc2[NH2+]C(=O)/C/1=N\NC(=O)c1ccc(cc1)Cl)OC(F)(F)F
**4f**	*N*'-[6-Trifluoromethoxy-2-oxo-1,2-dihydro-3*H*-indol-3-ylidene]-4-aminobenzohydrazide<br>c12ccc(cc2[NH2+]C(=O)/C/1=N\NC(=O)c1ccc(cc1)N)OC(F)(F)F
**4i**	*N*'-[6-Trifluoromethoxy-2-oxo-1,2-dihydro-3*H*-indol-3-ylidene]-4-hydroxybenzohydrazide<br>c12ccc(cc2[NH2+]C(=O)/C/1=N\NC(=O)c1ccc(cc1)O)OC(F)(F)F
**RM**	3-((2,6-Dichlorobenzylidene)hydrazono)indolin-2-one<br>O=C1Nc2ccccc2/C1=N/N=C/c1c(Cl)cccc1Cl
**RM2**	(2R)-2-[[6-(benzylamino)-9-propan-2-ylpurin-2-yl]amino]butan-1-ol (**Roscovitine**)<br>CC[C@H](CO)NC1=NC(=C2C(=N1)N(C=N2)C(C)C)NCC3=CC=CC=C3
**RM3**	2-[(2*S*)-1-[3-ethyl-7-[(1-oxidopyridin-1-ium-3-yl)methylamino]pyrazolo[1,5-a]pyrimidin-5-yl]piperidin-2-yl]ethanol (**Dinaciclib**)<br>CCC1=C2N=C(C=C(N2N=C1)NCC3=C[N+](=CC=C3)[O-])N4CCCC[C@H]4CCO

Table S2. Values of HOMO and LUMO energies, energy gap (ΔE_{GAP}), absolute hardness (η), chemical potential (μ), absolute softness (σ), global softness (S), absolute electronegativity (χ), global electrophilicity index (ω), and maximum additional electronic charge (ΔN_{MAX}).

Name	HOMO	LUMO	ΔE_{GAP}	η	μ	σ	s	χ	ω	ΔN_{MAX}
	[eV]	[eV]	[eV]	[eV]	[eV]	[eV^{-1}]	[eV^{-1}]	[eV]	[eV]	---
**1**	-6.49	-2.47	4.02	2.01	4.48	0.50	0.25	4.48	4.99	-2.23
**2a**	-6.48	-2.44	4.04	2.02	4.46	0.50	0.25	4.46	4.92	-2.21
**2b**	-6.54	-2.50	4.03	2.02	4.52	0.50	0.25	4.52	5.06	-2.24
**2c**	-6.51	-2.48	4.03	2.02	4.49	0.50	0.25	4.49	5.01	-2.23
**2d**	-6.53	-2.50	4.03	2.02	4.51	0.50	0.25	4.51	5.05	-2.24
**2e**	-6.53	-2.49	4.03	2.02	4.51	0.50	0.25	4.51	5.04	-2.24
**2f**	-5.95	-2.42	3.53	1.77	4.18	0.57	0.28	4.18	4.95	-2.37
**2h**	-6.55	-2.62	3.94	1.97	4.59	0.51	0.25	4.59	5.34	-2.33
**2i**	-6.45	-2.43	4.03	2.01	4.44	0.50	0.25	4.44	4.89	-2.20
**2j**	-6.44	-2.43	4.01	2.01	4.43	0.50	0.25	4.43	4.90	-2.21
**2k**	-6.53	-2.50	4.03	2.01	4.51	0.50	0.25	4.51	5.05	-2.24
**3a**	-6.48	-2.46	4.02	2.01	4.47	0.50	0.25	4.47	4.97	-2.22
**3b**	-6.52	-2.52	4.00	2.00	4.52	0.50	0.25	4.52	5.11	-2.26
**3c**	-6.51	-2.50	4.01	2.00	4.51	0.50	0.25	4.51	5.07	-2.25
**3d**	-6.52	-2.52	4.00	2.00	4.52	0.50	0.25	4.52	5.10	-2.26
**3e**	-6.52	-2.52	4.00	2.00	4.52	0.50	0.25	4.52	5.10	-2.26
**3f**	-6.03	-2.45	3.58	1.79	4.24	0.56	0.28	4.24	5.03	-2.37
**3g**	-5.57	-2.45	3.12	1.56	4.01	0.64	0.32	4.01	5.14	-2.57
**3h**	-6.54	-2.60	3.94	1.97	4.57	0.51	0.25	4.57	5.30	-2.32
**3i**	-6.47	-2.47	3.99	2.00	4.47	0.50	0.25	4.47	5.00	-2.24
**3j**	-6.46	-2.48	3.98	1.99	4.47	0.50	0.25	4.47	5.01	-2.24
**3k**	-6.22	-2.57	3.65	1.82	4.39	0.55	0.27	4.39	5.28	-2.41
**4a**	-6.17	-2.50	3.67	1.83	4.33	0.55	0.27	4.33	5.12	-2.36
**4b**	-6.22	-2.58	3.64	1.82	4.40	0.55	0.28	4.40	5.33	-2.42
**4c**	-6.19	-2.52	3.67	1.83	4.35	0.55	0.27	4.35	5.16	-2.37
**4d**	-6.20	-2.55	3.65	1.82	4.38	0.55	0.27	4.38	5.25	-2.40
**4e**	-6.20	-2.55	3.65	1.83	4.38	0.55	0.27	4.38	5.24	-2.40
**4f**	-6.01	-2.38	3.64	1.82	4.19	0.55	0.28	4.19	4.84	-2.31
**4g**	-5.62	-2.36	3.27	1.63	3.99	0.61	0.31	3.99	4.87	-2.44

	HOMO	LUMO
**1**	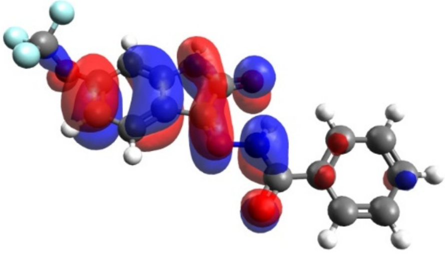	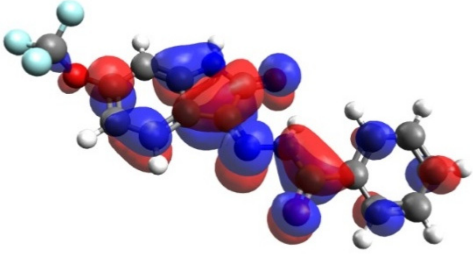
**2a**	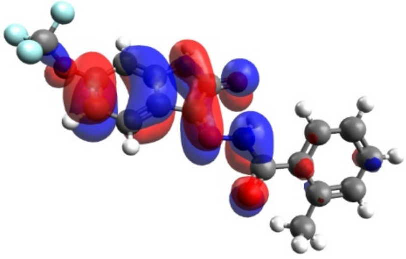	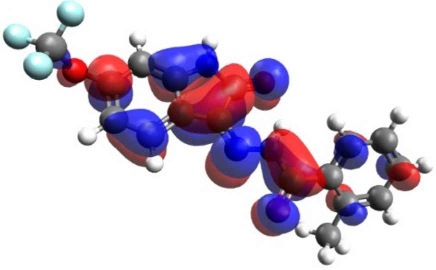
**2c**	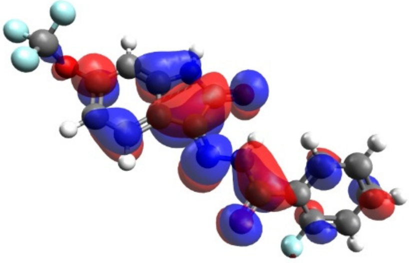	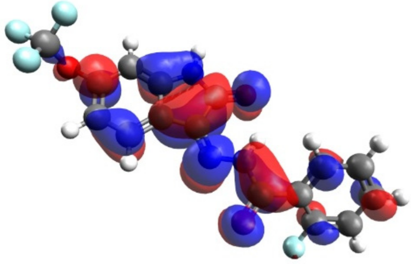
**2f**	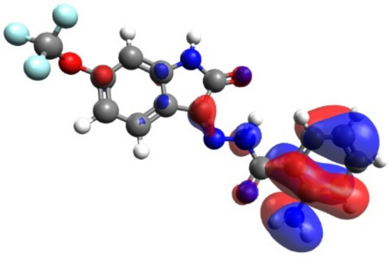	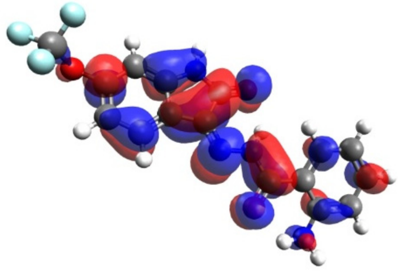
**2i**	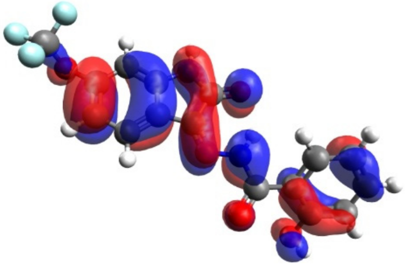	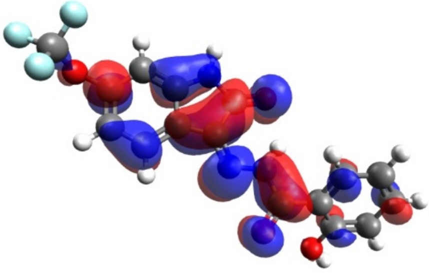
**3a**	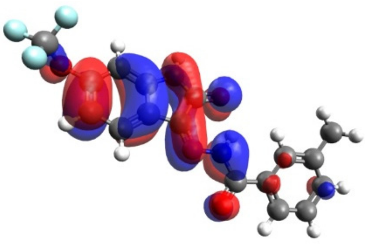	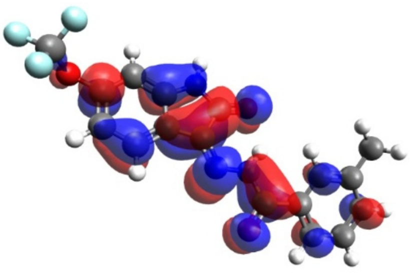

3b

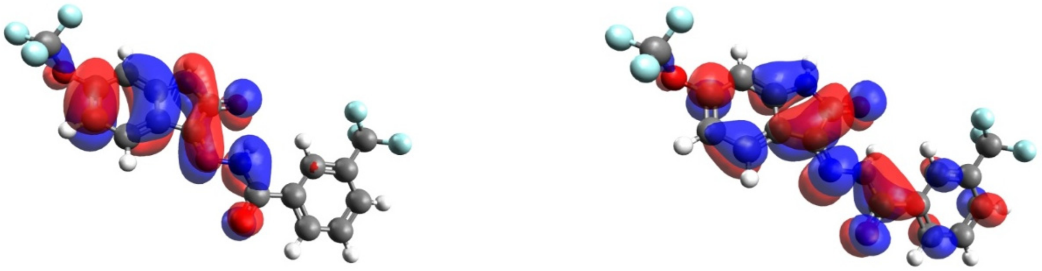

3c

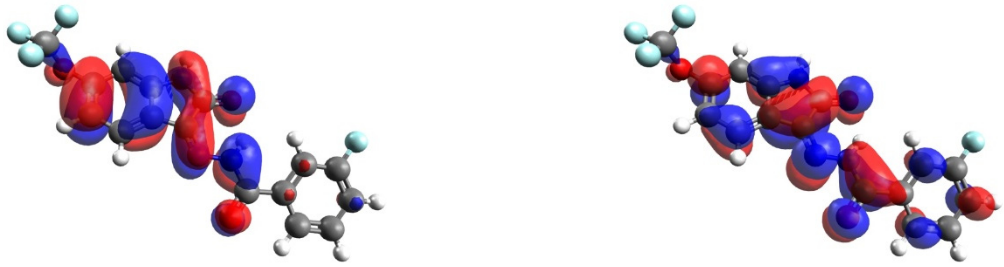

3d

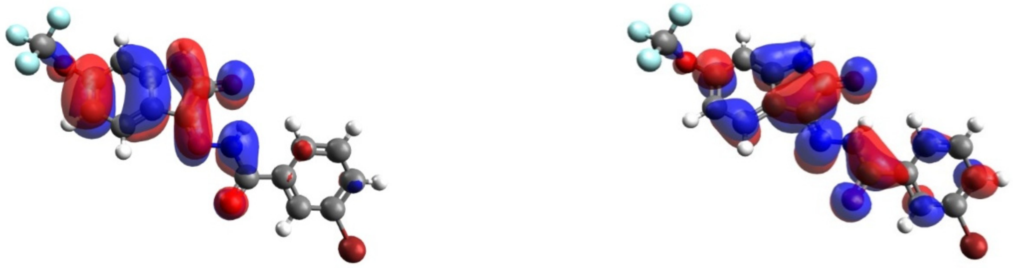

3e

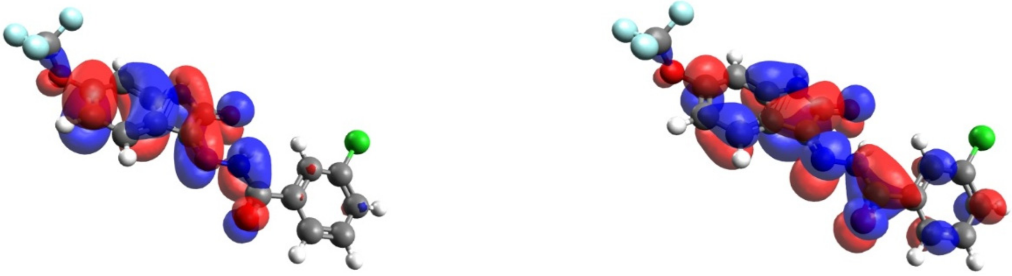

3h

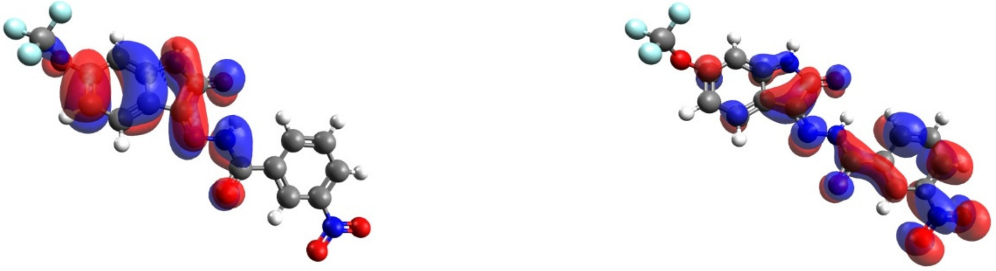

3k

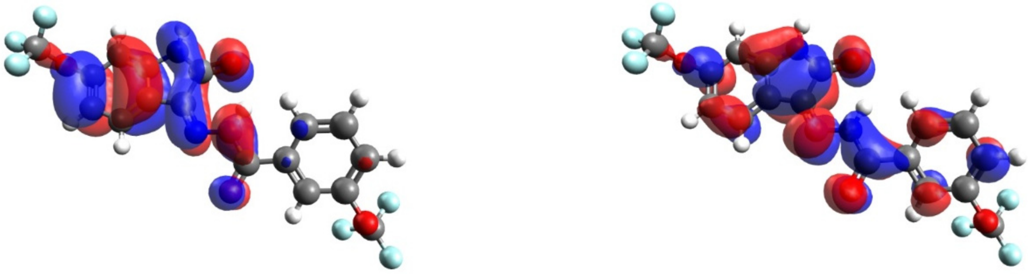

4a

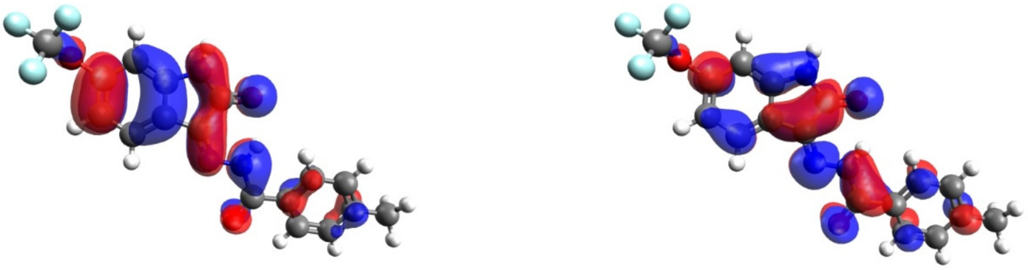

4b

4c

4d

4e

4f

4i

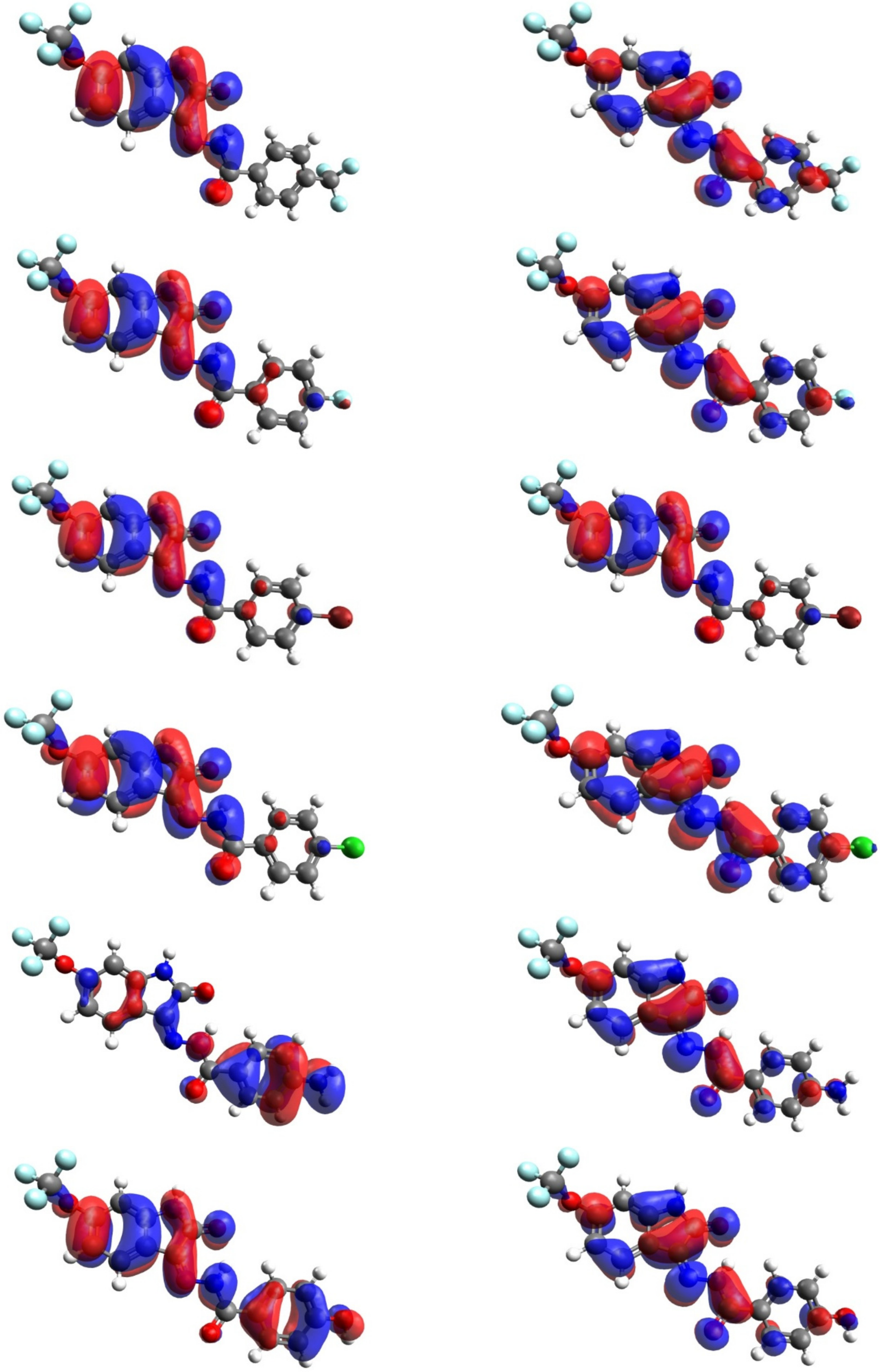

Figure S1. Graphical representation of the HOMO and LUMO orbital distributions of the studied 6-trifluoromethoxyisatin derivatives.

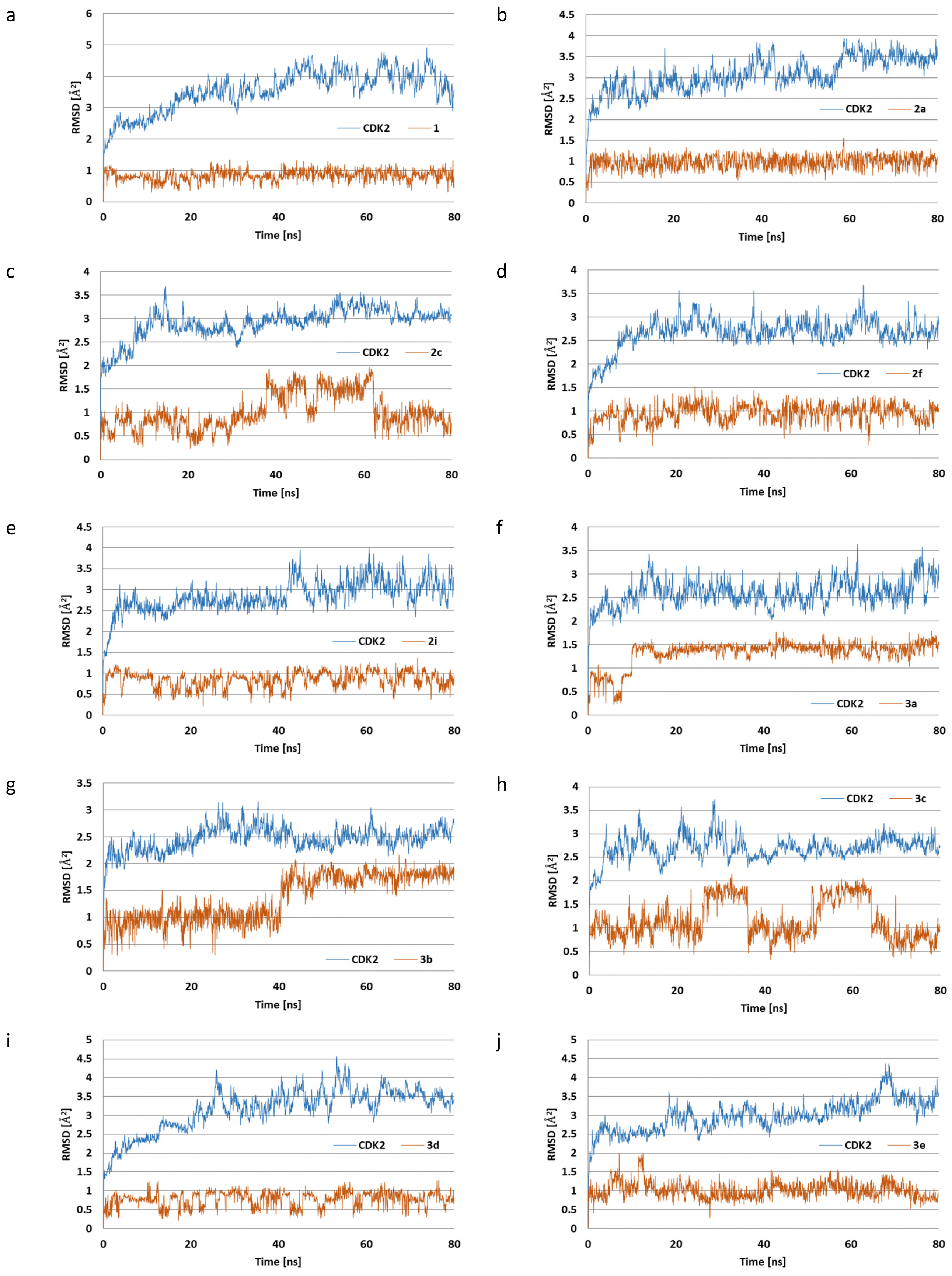

a
RMSD [Å²]
Time [ns]
CDK2
1
b
RMSD [Å²]
Time [ns]
CDK2
2a
c
RMSD [Å²]
Time [ns]
CDK2
2c
d
RMSD [Å²]
Time [ns]
CDK2
2f
e
RMSD [Å²]
Time [ns]
CDK2
2i
f
RMSD [Å²]
Time [ns]
CDK2
3a
g
RMSD [Å²]
Time [ns]
CDK2
3b
h
RMSD [Å²]
Time [ns]
CDK2
3c
i
RMSD [Å²]
Time [ns]
CDK2
3d
j
RMSD [Å²]
Time [ns]
CDK2
3e

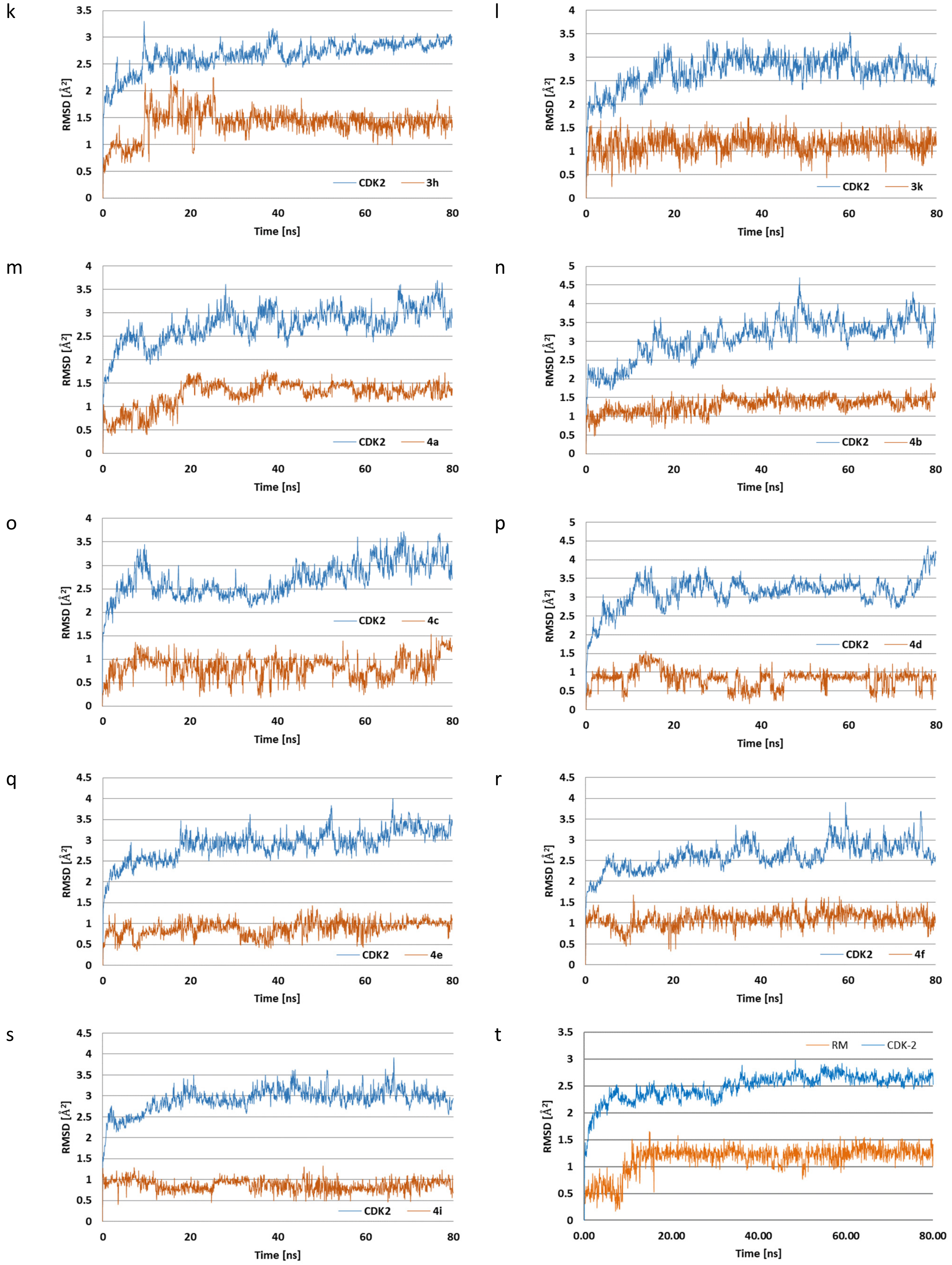

Figure S2. Distributions of RMSD values. Brown distributions refer to ligand molecules while blue distributions refer to CDK-2 protein.

Table S3. The cumulative analysis of the length of the interactions identified in CDK2 complexes with selected 6-Trifluoromethoxy-based benzoylhydrazides. The distances presented in the table represent middle values of intervals with a width of 0.2 Å.

INTERACTIONS	Population %								
	Σ	1.6 Å	1.8 Å	2 Å	2.2 Å	2.4 Å	2.6 Å	2.8 Å	3 Å
1									
Ligand (**H1**) … (**O**) GLU 81	100.0	1.6	41.0	44.5	11.2	1.4	0.3	0.1	0.0
Ligand (**O1**) … (**H**N) LEU 83	99.9	0.8	25.9	44.9	20.4	5.6	2.1	0.3	0.0
Ligand (**H5**) … (**O**) LEU 83	38.3	0.0	0.1	1.3	3.3	4.4	6.6	9.5	13.0
Ligand (**F**) … (**H**N) LYS 33	44.7	0.0	1.3	6.3	8.1	9.0	6.4	6.9	6.7
Ligand (**F**) … (**H**) ASP145	85.9	0.0	3.4	16.0	23.1	20.1	12.3	6.9	4.2
2a									
Ligand (**H1**) … (**O**) GLU 81	100.0	4.3	58.8	30.3	5.5	0.9	0.1	0.1	0.0
Ligand (**O2**) … (**H**N) LEU 83	100.0	0.5	26.8	46.0	19.6	5.6	1.0	0.4	0.2
Ligand (**H5**) … (**O**) LEU 83	7.3	0.0	0.1	0.1	0.8	1.1	0.6	1.8	2.9
2c									
Ligand (**H1**) … (**O**) GLU 81	100.0	2.1	40.9	42.8	11.8	2.0	0.3	0.1	0.0
Ligand (**O1**) … (**H**N) LEU 83	99.9	1.3	36.9	45.5	12.6	2.9	0.6	0.1	0.1
Ligand (**F**) … (**H**N) LYS 33	22.1	0.0	0.1	0.3	1.2	2.8	4.6	6.4	6.9
Ligand (**F**) … (**H**) ASP145	61.1	0.0	1.1	5.1	9.5	11.2	11.0	11.8	11.3
2f									
Ligand (**H1**) … (**O**) GLU 81	100.0	1.8	44.3	42.3	10.1	1.4	0.2	0.0	0.0
Ligand (**O1**) … (**H**N) LEU 83	100.0	0.4	36.6	42.3	15.7	3.6	1.0	0.4	0.1
Ligand (**H5**) … (**O**) LEU 83	51.8	0.1	2.1	9.7	8.8	7.2	6.4	7.7	9.9
Ligand (**F**) … (**H**N) LYS 33	38.7	0.0	0.0	0.5	2.3	5.4	9.1	11.3	10.1
Ligand (**F**) … (**H**) ASP145	60.5	0.1	1.5	6.8	9.8	11.1	10.8	10.3	10.2
2i									
Ligand (**H1**) … (**O**) GLU 81	100.0	1.5	43.9	44.8	8.6	1.1	0.1	0.1	0.0
Ligand (**O1**) … (**H**N) LEU 83	100.0	1.2	34.4	42.1	17.0	3.8	1.3	0.2	0.0
Ligand (**H5**) … (**O**) LEU 83	49.8	0.0	4.5	9.9	8.4	5.5	5.8	6.7	9.0
Ligand (**F**) … (**H**N) LYS 33	26.6	0.0	0.0	0.4	0.7	2.6	5.3	8.1	9.5
Ligand (**F**) … (**H**) ASP145	72.9	0.0	3.1	15.8	17.1	13.4	9.5	7.1	6.9
3a									
Ligand (**H1**) … (**O**) GLU 81	100.0	2.5	45.6	41.1	9.4	0.9	0.4	0.1	0.0
Ligand (**O1**) … (**H**N) LEU 83	100.0	0.6	36.1	43.2	14.8	4.6	0.6	0.0	0.1
Ligand (**H5**) … (**O**) LEU 83	72.0	0.1	3.4	13.9	13.6	9.6	10.4	9.0	11.9
Ligand (**F**) … (**H**N) LYS 33	23.6	0.0	0.1	0.3	0.6	2.9	5.6	6.8	7.4
Ligand (**F**) … (**H**) ASP145	54.3	0.0	0.6	5.4	9.3	9.8	8.3	10.7	10.2
3b									
Ligand (**H1**) … (**O**) GLU 81	100.0	1.2	39.3	45.6	11.3	2.0	0.4	0.2	0.0
Ligand (**O1**) … (**H**N) LEU 83	100.0	1.3	39.9	44.2	12.6	1.8	0.1	0.1	0.1
Ligand (**H5**) … (**O**) LEU 83	72.0	0.2	3.8	13.1	13.5	11.6	9.4	10.5	9.9
Ligand (**F**) … (**H**N) LYS 33	40.5	0.0	0.1	0.3	2.0	5.5	10.5	11.1	11.1
Ligand (**F**) … (**H**) ASP145	71.8	0.0	1.9	8.1	13.1	13.3	12.1	12.1	11.1
3c									
Ligand (**H1**) … (**O**) GLU 81	100.0	1.4	42.3	44.5	9.8	1.8	0.1	0.1	0.0
Ligand (**O1**) … (**H**N) LEU 83	100.0	1.3	43.3	42.1	11.5	1.5	0.2	0.1	0.0
Ligand (**H5**) … (**O**) LEU 83	57.9	0.0	2.5	7.9	9.4	9.6	7.9	10.4	10.0
Ligand (**F**) … (**H**N) LYS 33	24.5	0.0	0.0	0.3	0.8	2.9	5.4	6.9	8.1
Ligand (**F**) … (**H**) ASP145	79.2	0.0	1.6	8.6	16.5	17.2	14.0	12.5	8.7
3d									
Ligand (**H1**) … (**O**) GLU 81	100.0	1.7	41.4	43.4	11.3	1.9	0.2	0.1	0.0
Ligand (**O1**) … (**H**N) LEU 83	100.0	0.7	37.1	47.0	12.6	2.3	0.3	0.1	0.1
Ligand (**H5**) … (**O**) LEU 83	82.1	0.1	4.5	15.9	15.4	14.0	10.5	12.0	9.8
Ligand (**F**) … (**H**N) LYS 33	27.1	0.0	0.1	0.4	1.6	3.3	5.8	8.1	7.9
Ligand (**F**) … (**H**) ASP145	83.6	0.0	2.6	14.0	19.3	17.7	14.3	9.8	5.9
3e									
Ligand (**H1**) … (**O**) GLU 81	100.0	2.1	47.4	39.8	9.6	0.9	0.3	0.0	0.0
Ligand (**O1**) … (**H**N) LEU 83	99.8	0.8	38.9	44.8	12.9	2.1	0.4	0.1	0.0
Ligand (**H5**) … (**O**) LEU 83	80.2	0.1	7.1	24.1	17.2	11.5	7.8	5.9	6.4
Ligand (**F**) … (**H**N) LYS 33	45.8	0.0	0.0	1.2	3.7	7.3	11.0	12.1	10.6
Ligand (**F**) … (**H**) ASP145	39.3	0.0	0.1	1.7	4.4	5.8	7.3	10.0	9.9
3h									
Ligand (**H1**) … (**O**) GLU 81	100.0	2.6	47.6	41.3	7.4	1.0	0.0	0.0	0.0
Ligand (**O1**) … (**H**N) LEU 83	100.0	1.3	34.7	46.4	14.3	2.9	0.4	0.1	0.0
Ligand (**H5**) … (**O**) LEU 83	84.1	0.0	0.0	0.0	1.8	20.1	28.6	20.2	13.5
Ligand (**F**) … (**H**N) LYS 33	34.1	0.0	0.1	0.3	1.1	3.8	7.8	10.3	10.8
Ligand (**F**) … (**H**) ASP145	95.2	0.3	10.1	26.6	25.0	15.8	9.8	4.9	2.8
3k									
Ligand (**H1**) … (**O**) GLU 81	100.0	1.3	47.9	41.6	8.3	0.9	0.1	0.0	0.0

Ligand (**O1**) … (**H**N) LEU 83	100.0	1.1	41.3	45.0	10.8	1.6	0.4	0.0	0.0
Ligand (**H5**) … (**O**) LEU 83	78.3	0.0	2.8	13.6	14.4	14.2	11.6	12.2	9.4
Ligand (**F**) … (**H**N) LYS 33	45.9	0.0	0.1	0.7	2.9	6.9	10.6	12.5	12.3
Ligand (**F**) … (**H**) ASP145	83.7	0.0	2.4	11.7	16.8	16.5	13.8	13.8	8.8
4a									
Ligand (**H1**) … (**O**) GLU 81	100.0	1.3	42.5	44.1	10.5	1.4	0.3	0.0	0.0
Ligand (**O1**) … (**H**N) LEU 83	100.0	1.2	38.5	44.3	12.8	2.6	0.5	0.1	0.0
Ligand (**H5**) … (**O**) LEU 83	69.1	0.1	1.8	7.6	11.4	10.4	14.0	12.1	11.8
Ligand (**F**) … (**H**N) LYS 33	26.8	0.0	0.1	0.6	2.4	2.8	6.6	7.0	7.4
Ligand (**F**) … (**H**) ASP145	80.2	0.0	1.6	10.5	17.3	20.8	13.9	10.1	5.9
4b									
Ligand (**H1**) … (**O**) GLU 81	100.0	1.1	40.1	46.3	10.3	1.8	0.3	0.1	0.0
Ligand (**O1**) … (**H**N) LEU 83	100.0	0.8	38.6	46.8	10.9	2.4	0.4	0.0	0.0
Ligand (**H5**) … (**O**) LEU 83	82.6	0.0	5.4	19.5	17.6	14.1	11.0	7.8	7.3
Ligand (**F**) … (**H**N) LYS 33	27.6	0.0	0.1	0.4	1.3	4.6	5.9	7.6	7.8
Ligand (**F**) … (**H**) ASP145	56.8	0.0	0.8	6.7	10.9	10.3	10.8	8.6	8.8
4c									
Ligand (**H1**) … (**O**) GLU 81	100.0	1.0	42.4	44.4	10.6	1.4	0.3	0.0	0.0
Ligand (**O1**) … (**H**N) LEU 83	100.0	0.6	32.3	45.6	15.8	4.3	1.1	0.2	0.1
Ligand (**H5**) … (**O**) LEU 83	54.4	0.0	2.5	8.4	10.3	9.4	7.9	7.6	8.3
Ligand (**F**) … (**H**N) LYS 33	19.8	0.0	0.0	0.1	0.6	2.3	3.8	6.3	6.8
Ligand (**F**) … (**H**) ASP145	43.8	0.0	0.2	1.6	3.7	7.1	8.9	10.7	11.6
4d									
Ligand (**H1**) … (**O**) GLU 81	100.0	1.9	45.3	41.3	9.3	1.6	0.3	0.3	0.0
Ligand (**O1**) … (**H**N) LEU 83	100.0	0.7	32.3	44.8	17.4	3.5	1.2	0.1	0.0
Ligand (**H5**) … (**O**) LEU 83	43.3	0.0	0.4	2.6	3.9	5.3	8.4	9.6	12.9
Ligand (**F**) … (**H**N) LYS 33	38.1	0.0	0.0	0.7	2.2	5.5	8.8	10.8	10.1
Ligand (**F**) … (**H**) ASP145	76.8	0.0	2.1	10.4	17.1	17.5	14.2	8.9	6.5
4e									
Ligand (**H1**) … (**O**) GLU 81	100.0	1.1	39.3	46.5	11.1	1.4	0.5	0.1	0.1
Ligand (**O1**) … (**H**N) LEU 83	100.0	0.9	33.6	45.4	15.4	4.0	0.6	0.1	0.0
Ligand (**H5**) … (**O**) LEU 83	42.0	0.0	0.7	5.5	5.5	6.3	7.1	7.8	9.2
Ligand (**F**) … (**H**N) LYS 33	32.7	0.0	0.0	0.5	1.9	4.1	7.3	8.6	10.3
Ligand (**F**) … (**H**) ASP145	61.8	0.0	3.4	8.7	9.4	10.8	9.5	9.5	10.6
Ligand(**O1**) … (**H5**) Ligand	96.4	0.0	0.0	3.6	20.6	33.2	20.8	12.6	5.6
4f									
Ligand (**H1**) … (**O**) GLU 81	100.0	1.7	42.6	43.9	10.1	1.3	0.3	0.1	0.0
Ligand (**O1**) … (**H**N) LEU 83	100.0	0.9	31.1	45.9	17.0	4.1	0.7	0.2	0.1
Ligand (**H5**) … (**O**) LEU 83	41.1	0.0	0.6	4.2	6.3	6.5	5.9	8.4	9.2
Ligand (**F**) … (**H**N) LYS 33	24.6	0.0	0.0	0.4	0.7	2.4	5.2	7.0	8.8
Ligand (**F**) … (**H**) ASP145	87.4	0.0	4.3	18.4	23.3	18.8	11.2	6.4	5.1
4i									
Ligand (**H1**) … (**O**) GLU 81	100.0	1.3	44.8	44.0	8.9	0.9	0.2	0.0	0.0
Ligand (**O1**) … (**H**N) LEU 83	100.0	1.3	37.6	45.0	12.5	3.0	0.5	0.1	0.1
Ligand (**H5**) … (**O**) LEU 83	65.4	0.3	3.6	10.3	12.0	9.8	9.0	9.3	11.2
Ligand (**F**) … (**H**N) LYS 33	37.1	0.0	0.0	0.8	1.9	5.4	8.0	10.4	10.7
Ligand (**F**) … (**H**) ASP145	48.4	0.0	0.4	2.8	5.9	9.6	9.4	9.8	10.6
RM									
Ligand (**H1**) … (**O**) GLU 81	99.94	1.31	39.44	44.25	12.94	1.75	0.25	0.00	0.00
Ligand (**O1**) … (**H**N) LEU 83	98.94	0.13	5.69	23.81	27.25	19.75	12.06	7.13	3.13
